# Adult-Onset Anti-Citrullinated Peptide Antibody-Negative Destructive Rheumatoid Arthritis Is Characterized by a Disease-Specific CD8+ T Lymphocyte Signature

**DOI:** 10.3389/fimmu.2020.578848

**Published:** 2020-11-19

**Authors:** Tiina Kelkka, Paula Savola, Dipabarna Bhattacharya, Jani Huuhtanen, Tapio Lönnberg, Matti Kankainen, Kirsi Paalanen, Mikko Tyster, Maija Lepistö, Pekka Ellonen, Johannes Smolander, Samuli Eldfors, Bhagwan Yadav, Sofia Khan, Riitta Koivuniemi, Christopher Sjöwall, Laura L. Elo, Harri Lähdesmäki, Yuka Maeda, Hiroyoshi Nishikawa, Marjatta Leirisalo-Repo, Tuulikki Sokka-Isler, Satu Mustjoki

**Affiliations:** ^1^Hematology Research Unit Helsinki, University of Helsinki, Helsinki, Finland; ^2^Department of Hematology, Helsinki University Hospital Comprehensive Cancer Center, Helsinki, Finland; ^3^Department of Clinical Chemistry and Hematology, University of Helsinki, Helsinki, Finland; ^4^Translational Immunology Research Program, University of Helsinki, Helsinki, Finland; ^5^Turku Bioscience Centre, University of Turku and Åbo Akademi University, Turku, Finland; ^6^Rheumatology, Jyväskylä Central Hospital, Jyväskylä, Finland; ^7^Institute for Molecular Medicine Finland (FIMM), Helsinki Institute of Life Science (HiLIFE), University of Helsinki, Helsinki, Finland; ^8^Rheumatology, University of Helsinki, Helsinki University Hospital, Helsinki, Finland; ^9^Department of Biomedical and Clinical Sciences, Division of Inflammation and Infection, Linköping University, Linköping, Sweden; ^10^Institute of Biomedicine, University of Turku, Turku, Finland; ^11^Department of Computer Science, Aalto University School of Science, Espoo, Finland; ^12^Division of Cancer Immunology, Research Institute/Exploratory Oncology Research and Clinical Trial Center (EPOC), National Cancer Center, Tokyo, Japan; ^13^University of Eastern Finland, Faculty of Health Sciences, Kuopio, Finland

**Keywords:** rheumatoid arthritis, seronegative, ACPA-negative, CD8+ lymphocyte, T cell receptor, somatic mutation

## Abstract

Rheumatoid arthritis (RA) is a complex autoimmune disease targeting synovial joints. Traditionally, RA is divided into seropositive (SP) and seronegative (SN) disease forms, the latter consisting of an array of unrelated diseases with joint involvement. Recently, we described a severe form of SN-RA that associates with characteristic joint destruction. Here, we sought biological characteristics to differentiate this rare but aggressive anti-citrullinated peptide antibody-negative destructive RA (CND-RA) from early seropositive (SP-RA) and seronegative rheumatoid arthritis (SN-RA). We also aimed to study cytotoxic CD8+ lymphocytes in autoimmune arthritis. CND-RA, SP-RA and SN-RA were compared to healthy controls to reveal differences in T-cell receptor beta (TCRβ) repertoire, cytokine levels and autoantibody repertoires. Whole-exome sequencing (WES) followed by single-cell RNA-sequencing (sc-RNA-seq) was performed to study somatic mutations in a clonally expanded CD8+ lymphocyte population in an index patient. A unique TCRβ signature was detected in CND-RA patients. In addition, CND-RA patients expressed higher levels of the bone destruction-associated TNFSF14 cytokine. Blood IgG repertoire from CND-RA patients recognized fewer endogenous proteins than SP-RA patients’ repertoires. Using WES, we detected a stable mutation profile in the clonally expanded CD8+ T-cell population characterized by cytotoxic gene expression signature discovered by sc-RNA-sequencing. Our results identify CND-RA as an independent RA subset and reveal a CND-RA specific TCR signature in the CD8+ lymphocytes. Improved classification of seronegative RA patients underlines the heterogeneity of RA and also, facilitates development of improved therapeutic options for the treatment resistant patients.

## Introduction

Rheumatoid arthritis is a chronic autoimmune disease in which the patient’s own immune system targets the synovial tissue in joints. According to the American College of Rheumatology (ACR) and European League Against Rheumatism (EULAR) classification criteria for rheumatoid arthritis (RA), seropositive RA (SP-RA) is characterized by the presence of anti-cyclic citrullinated peptide antibodies (ACPA) and/or rheumatoid factor (RF). Seronegative RA (SN-RA) is diagnosed when the diagnostic autoantibodies are not present and other underlying causes of joint inflammation have been excluded (1). In practice, SN-RA diagnosis is unspecific, and most patients can be reassigned to some other diagnosis during the follow-up (2).

We have recently provided a clinical description of an unusually severe form of SN-RA (3). These patients are negative for the clinically used anti-cyclic citrullinated peptide 2 (CCP2) test, and the clinical manifestations include the progression of joint destruction despite active therapy. Thus, we named the cohort as CCP-negative destructive (CND) RA, and differently from the classical SP-RA, the destruction is predominantly localized in the wrists, ankles, and even in large joints (3). The biomedical characteristics of this RA sub-type have not been studied before.

While SP-RA is strongly associated with the MHC II coding *HLA-DRB1* (4), the genetic predisposition for SN-RA is different (5–7). The profound differences in disease genetics imply that there are also fundamental differences in disease pathomechanism between SP-RA and SN-RA. As many of the disease-associated loci especially in SN-RA are localized within the HLA-I loci, the role of CD8+ lymphocytes in RA should be investigated in more detail.

Replication errors are a natural part of DNA replication. Antigen recognition and the following T-cell activation and proliferation predispose the activated cells’ genomes to somatic mutations. The instrumental role of somatic mutations as drivers of cancer has been documented in detail (8). On the other hand, somatic mutations also accumulate in healthy tissues (9) with the highest mutation burdens reported thus far in the skin (10, 11), lung (12), and esophageal epithelium (13, 14).

Some of the mutations, especially in white blood cells, may contribute to chronic inflammation and autoimmunity (15–17). This hypothesis is supported by the identification of somatic mutations in the circulating, cytotoxic CD8+ and CD4+ lymphocytes in RA, Felty’s syndrome, aplastic anemia, multiple sclerosis, chronic graft-versus-host-disease (cGVHD) and common variable immune deficiency patients (16–22). The mutation harboring CD8+ lymphocyte clones in RA are stable, cytotoxic effector memory cells (16). Somatic mutations may modulate the function of mutation carrying T-cells, and thereby participate in disease progression.

We here present biological evidence that CND-RA is an independent subgroup of SN-RA with a unique CD8+ lymphocyte signature and a narrower autoantibody repertoire than diagnostic SP-RA.

## Materials and Methods

For a comprehensive description see the **Supplementary Material**.

### Ethical Approval, Public Involvement, and Sample Collection

The study was approved by the Helsinki University Hospital Ethics Committee. Patients and healthy controls were enrolled to the study in the Departments of Rheumatology at Jyväskylä Central Hospital and Helsinki University Hospital. After the study was introduced, all patients signed written informed consent. We followed the guidelines of the Declaration of Helsinki and recorded routine laboratory and clinical parameters at the time of sampling (**Table 1** and in **Supplementary Tables 1** and **2****)**.

**Table 1:** Patients’ characteristics.

Patient	Age at dg	Age at sampling	ESR (mm/h)	CRP (mg/l)	RF (IU/ml)	ACPA (U/ml)	aCARB	HLA-B27	Treatment at sampling
Pt-1	45–55	65–75	24	3	<30	<7	neg	+	ADA, MTX
Pt-2	25–35	55–65	23	3	<30	<7	neg	+	ETA, ABA, MTX
Pt-3	25–35	55–65	19	3	<30	<7	neg	−	INF, MTX, PRED
Pt-4	15–25	35–45	5	0	<30	<7	neg	−	ETA, MTX, PRED
Pt-5	55–65	55–65	42	109	<30	<7	ND	−	INF, MTX, PRED
Pt-6	45–55	55–65	8	1	<30	<7	ND	−	ETA, MTX, HXC
Pt-7	45–55	65–75	8	1	32	8	ND	−	ABA, MTX, PRED
Pt-8	25–35	55–65	9	2	37	<7	ND	−	HXC, PRED

Age at dg, age at diagnosis expressed as 10-year age intervals; ESR, erythrocyte sedimentation rate; CRP, C-reactive protein; RF, rheumatoid factor; ACPA, anti-cyclic citrullinated peptide antibodies; aCARB, anti-carbamylated protein antibodies; ND, not done; HLA-B27, presence of the HLA-B27 allele; ADA, adalimumab; MTX, methotrexate; ETA, etanercept; ABA, abatasept; INF, infliximab; PRED, prednisolon; HXC, hydroxycloroquine.

Peripheral blood samples (50 ml EDTA blood) were collected for the study. Buffy coats from healthy controls were provided by the Histocompatibility Testing Laboratory, Finnish Red Cross Blood service. Peripheral blood mononuclear cells (PBMNCs) were separated from EDTA blood or buffy coats using Ficoll gradient separation (Ficoll-Paque PLUS, GE Healthcare).

### Flow Cytometry

Peripheral blood samples were stained for lymphocyte clonality analysis with anti-CD3 (SK7), anti-CD4 (SK3), and anti-CD8 (SK-1) (Becton Dickinson) and a panel of T-cell receptor β variable chain (TCR Vβ) antibodies (IOTest Beta Mark TCR V kit, Beckman Coulter Immunotech, cat. no IM3497), which recognize approximately 70% to 80% of the human TCR β V regions. After staining, red blood cells were lysed with BD FACS Lysing Solution (Becton Dickinson Biosciences) and re-suspended to phosphate-buffered saline (PBS) with 2 mM EDTA. Samples were acquired with FACSAria II or FACS Verse (Becton Dickinson) and analyzed with FlowJo software (Becton Dickinson).

Cell sorting was performed either from fresh or from cryopreserved PBMCs using FACSAria II (Becton Dickinson) or Sony SH800 (Sony Biotechnology Inc.). For sorting, MNCs were stained with anti-CD3 (SK7), anti-CD4 (SK3), anti-CD8 (SK-1), and the appropriate anti-Vβ antibody from the IOTest Beta Mark TCR V kit. Cell fractions’ purities were controlled with flow cytometry, and the purities of all sorted fractions were nearly 100%.

### T-Cell Receptor Beta Repertoire Analysis

T-cell receptor beta (TCRβ) sequencing was performed from CD8+ cells’ genomic DNA according to the manufacturer’s instructions. Sequencing and data analysis were conducted as previously described in the FIMM Technology center with ImmunoSEQ assay by Adaptive Biotechnologies Corp. SP-RA, SN-RA and healthy controls have already been reported previously (16).

The analyses were started with the TCRβ matrices provided by the Adaptive Biotechnologies preprocessing pipeline. All data were transformed to VDJtools (23) format to reduce data complexity. Non-productive clonotypes were removed from the analysis. To assess the saturation of the sequencing results between the cohorts, the dependencies between samples diversity and sample size were determined with rarefaction plots inspired by Colwell et al. (24), and as implemented in VDJtools. We used a minimum sampling depth of 10 000 reads per sample and subsampled all samples to 10 000 reads. This normalization was performed to remove statistical biases for depth-dependent parameters. Multiple different diversity metrics, including Shannon-Wiener, Simpson and clonality indexes, were calculated with the CalcDiversityStats function.

For the subsampled data, we analyzed the antigen-specificities of non-singleton TCRβs, i.e. clonotypes with at least two supporting reads. To analyze the antigen-specificities of TCRβs in a supervised manner, we used VDJdb (25), the largest repository of TCR-sequences with known specificity to hard-match (=no mismatches allowed) against the TCRβ-sequences in our data. To analyze the TCR antigen-specificities in an unsupervised manner, we analyzed the amino acid mismatches (Hamming distance) within the antigen binding CDR3 part (26). TCRs that differed by one amino acid were pooled together to form a cluster with a potentially shared antigen target.

For the subsampled data, public TCRs across the samples were pooled together with the JoinSamples-command at amino acid level. Publicness was defined as clonotypes that were shared by at least two individuals. To normalize the sample numbers, subgroups were randomly resampled to yield the same number of samples (n=6 for each), and this was iterated ten times. The statistically enriched clonotypes were calculated with one-sided Fisher’s exact test, and the threshold for enrichment was chosen as uncorrected p < 0.05. After p-value correction (Benjamini-Hochberg), none of the 117 399 tested clonotypes remained significant.

### Autoantibody Screen and Analysis

Autoantibodies were analyzed using the Invitrogen (Carlsbad, California, USA) ProtoArray protein microarray v5.1 platform. To compare the numbers of autoreactive antibodies in CND-RA, SP-RA and in the healthy controls, the cut-off for positivity was set to fold change 10 (FC10) compared to the average of healthy controls (n=5). The cutoff of FC15 was used for illustrations.

### Cytokine Profiling

EDTA plasma samples were analyzed with Proseek Multiplex Inflammation I (Olink Biosciences) immunoassay as previously described (18). The panel includes the simultaneous measurement of 92 inflammation-related human proteins.

### Variant Identification

Calling of somatic variants from whole-exome sequencing data was performed similar to our previous study (17) and included mapping reads against GRCh38 reference with BWA (27) and calling variants with the GATK toolkit and MuTect2 (28).

### sc-RNA-Sequencing (sc-RNA-Seq)

Magnetic bead separated total CD8+ cells and flow-cytometry enriched Vb22 expressing clonal cells (gating strategy and purity plots are presented in **Supplementary Figure 1**) were partitioned using the Chromium Controller (10x Genomics), aiming at the capture of 6000 single cells from each sample. The sequencing libraries were generated using the single cell 3´ reagent kit (V1), following the manufacturer’s instructions. The libraries were sequenced using HiSeq 2500 (Illumina). The median sequencing depths were 5734 (bead-separated), and 7575 reads per cell (flow-enriched). The raw sequencing data were processed using CellRanger pipeline version 1.2.0 (10x Genomics) using the GRCh38 reference genome. The R single-cell toolkit SEURAT (29) version 2.3.4 was used for the analysis of single-cell transcriptomics data.

The single-cell gene expression matrices corresponding to the magnetic bead separated total CD8+ cells and flow-cytometry enriched Vb22 expressing clonal cells were merged into one Seurat object for downstream analysis. Dimensionality reduction for the data set was performed using PCA (with the top 2000 highly variable genes as per Seurat guidelines) and top 12 PCs were selected. Clusters were determined with a resolution of. 6 and tSNE was used (k=30) for visualization.

Additionally, Canonical Correlation analysis (CCA) was used to align (and cluster) the two data sets and to see common sources of variations between the data sets (**Supplementary Materials and Methods, Supplementary Figures 2** and **3**).

### Data Sharing and Code Availability

TCR sequencing data are available in ImmuneAccess with the identifiers: https://doi.org/10.21417/B76C7W and (added once accepted). Other sequence data (immunogene panel, exome, and sc-RNA-sequencing) are available from corresponding authors upon request, owing to regulations pertaining to the authors’ ethics permit. Code related to TCRβ repertoire analysis can be found in https://github.com/janihuuh/snd_ra_tcrb_manu.

### Statistical Analyses

Statistical analyses were performed using Prism 6, Qlucore Omics Explorer (Qlucore AB, Lund, Sweden) and R. In all analyses, p-values < 0.05 were considered as statistically significant. All statistical methods are given in figure legends. * p-values < 0.05, ** p-values < 0.01, *** p-values 0.001.

## Results

### Patients’ Characteristics

All CND-RA patients had developed typical joint destructions in wrists and ankles and had failed to respond to conventional anti-rheumatic therapy. Seven out of eight (n=8) included patients were on biological drugs at sampling, and detailed clinical characteristics can be found in **Table 1** and **Supplementary Table 1**. SP-RA (n=51), SN-RA (n=11) patients, and the healthy controls (n=28) have been previously described in (16), and their characteristics are now summarized in **Supplementary Table 2**.

### CND-RA Patients Harbor Fewer Antibodies Against Endogenous Proteins Than Patients With SP-RA

**Figure 1:**
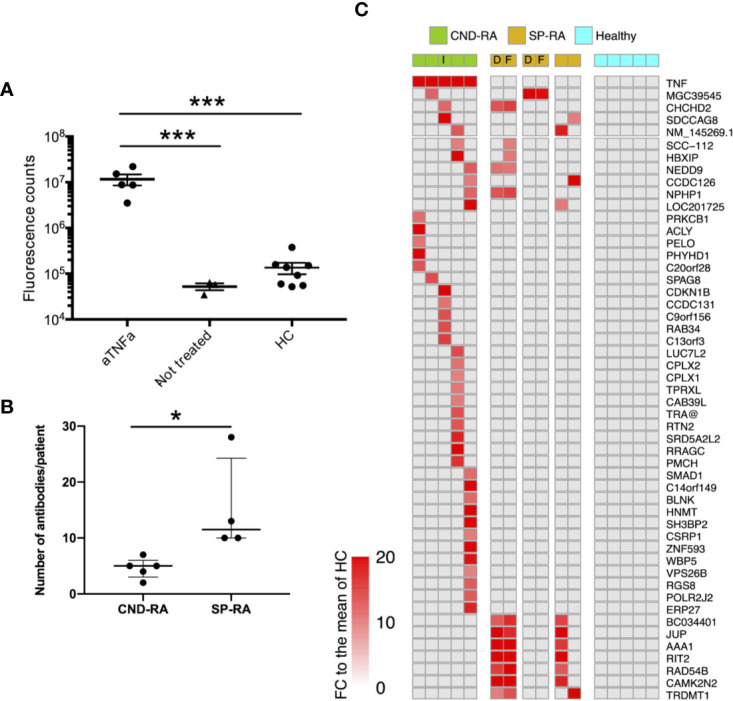
**(A)** Anti-TNFa antibodies were analyzed from plasma samples using a DELFIA based sandwich immunoassay. Statistics were calculated using One-way ANOVA with Tukey’s post-hoc test. **(B)** Number of positive autoantibody findings in CND-RA and diagnostic phase SP-RA samples. Samples were regarded as positive when the observed fluorescence intensity exceeded fold change (FC) 10 compared to the mean of healthy controls. Median values are presented with inter-quartile ranges, Unpaired T-test. **(C)** Heat-map illustrating all antibodies that were detected in CND-RA. In addition, antibodies that were identified in more than one SP-RA samples are presented in the lower panel. Healthy controls did not have any reported autoantibodies. The cut-off for positivity is FC 15 compared to the mean of HC. aTNFa, anti-tumor necrosis factor-alpha; HC, healthy controls; CND-RA, ACPA-negative destructive rheumatoid arthritis; SP-RA, seropositive rheumatoid arthritis; D, diagnostic sample; T, sample from a treated patient; I, index patient for somatic mutation and sc-RNA-seq analyses. *p < 0.05, ***p < 0.001.

Antibody repertoires against endogenous proteins were analyzed using ProtoArray protein microarray, and assay performance was validated using a DELFIA assay that detected the therapeutic anti-TNFa antibodies in the CND-RA samples (**Figure 1A**).

CND-RA patients with decades of disease history displayed fewer antibodies against endogenous proteins than the diagnostic SP-RA patients (**Figure 1B**). **Figure 1C** shows all autoreactive antibodies (FC>15 to the mean of healthy controls) that were detected in CND-RA together with autoreactive antibodies that were detected in at least 2 SP-RA patients. All RA-associated antibodies identified in the microarray experiment, including the previously reported anti-ZNF706 autoantibody (30) are shown in the **Supplementary Figure 4**. The reported antibodies were not detected in any of the healthy controls (n=5). Antibody profiles were stable as paired samples from two SP-RA patients after 14- or 16-months follow-up resulted in highly comparable results (**Figure 1A** and **Supplementary Figure 4**).

### T-Cell Receptor Signature Differentiates CND-RA From SN-RA, SP-RA, and Healthy Controls

Each T-cell recombines its T-cell receptor (TCR) locus during thymic maturation. The recombined TCR is unique for each naïve T-cell, and all T-cells with identical TCRs are almost certainly derived from one single cell. TCR is responsible for antigen recognition in the context of either HLA I (CD8+ lymphocytes) or HLA II (CD4+ lymphocytes). As our patient cohort differed from the HLA II-associated seropositive RA, we hypothesized that this difference might translate into a unique CD8+ TCR signature. Similarly, as previously shown in SP-RA (16), CD8+ lymphocytes were more clonal than CD4+ lymphocytes in CND-RA (**Supplementary Figure 5**).

We compared the CD8+ TCRβ repertoires of CND-RA patients (n=8) to the TCRβ repertoires of newly diagnosed patients with either SP-RA (n=51) or SN-RA (n=11). To avoid sequencing depth induced bias in TCR diversity metrics, we selected a cut-off of 10 000 reads and removed samples below this. All samples passing the cut-off value (CND-RA n=6, SN-RA n=7, SP-RA n=46 and HC n=28) were resampled to 10 000 reads before further data analyses.

**Figure 2:**
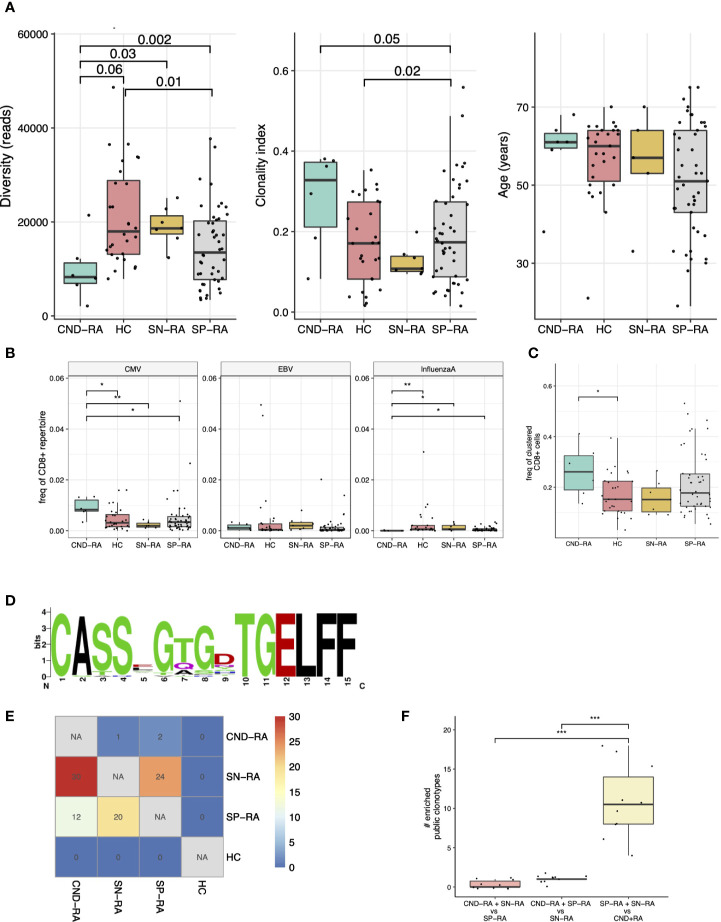
T-cell receptor beta (TCRβ) repertoire analysis of CND-RA patients. **(A)** TCRβ repertoire diversity (left) and clonality index (middle) were analyzed to reveal differences in sample clonality. There was no statistically significant difference in age between the groups (right). **(B)** Pooled frequency of cytomegalovirus (CMV), Epstein-Barr virus (EBV) or influenza A epitope-specific TCRs. Epitope-specificities were determined as hard-matches against VDJdb-database, the biggest repository of TCRs with known epitope-specificities. **(C)** Frequency of TCRβ sequences that could be assigned to potentially epitope-specific clusters. Potentially epitope-specific clusters were determined as TCRs with one amino acid mismatch (Hamming distance = 1). **(D)** A logoplot showing the amino acid sequence of the CDR3s in one representative epitope-specific cluster with the highest mean frequency among CND-RA patients compared to other subgroups (SN-RA, SP-RA and HC). **(E)** Heatmap showing the median amount of enriched (Fisher’s exact test, one-sided) public clonotypes to subgroups after resampling the groups 10 times to the same sample size (n=6). These enriched public clonotypes could be more important to disease pathogenesis than public clonotypes that are not enriched to any subgroup. **(F)** Numbers of enriched public clonotypes in group combinations after the same resampling as in E. In A-C: CND-RA n=6, SN-RA n=7, SP-RA n=46, HC n=28. All tests **(A–F)** with the Mann-Whitney test unless otherwise stated. *p < 0.05, **p < 0.01, ***p < 0.001.

Clonality metrics are known to correlate with age, and this was also seen in our data set (**Supplementary Figure 6****)**. By using a linear regression model with age as confounding factor, we discovered that CND-RA patients had less diverse, i.e. more clonal CD8+ TCRβ-repertoire than the other subtypes of RA (**Figure 2A**). There were no statistically significant differences in age between the groups.

As many autoimmune disorders are associated with viral infections, we hard-matched (searched for completely matching sequences) the TCRβ sequences from the patient samples against VDJdb, currently the biggest TCR data repository with annotated epitope-specificities (25). We detected an increased abundance of cytomegalovirus (CMV) reactive T-cells but a decreased abundance of influenza-A reactive CD8+ cells in CND-RA when compared to healthy controls and other RA subtypes (**Figure 2B**). The antigens with the most significant variation were CMV IE-1 and influenza A M1 (**Supplementary Figure 7**).

We (31) and others (32, 33) have recently shown that structurally similar TCRs are likely to bind similar antigens. Thus, to find T-cell specificities against previously unknown antigens, we grouped TCRs with one amino acid mismatch into potentially epitope-specific clusters. After pooling all samples, we found 631 clusters altogether. CND-RA patients had the highest proportion of clustered CD8+ T cells (the proportion of CD8+ belonging to any of the clusters), and this finding was statistically significant against healthy controls but not against other RA subtypes (**Figure 2C**). Interestingly, while CND-RA patients had the highest abundance of the clustered CD8+ cells, it had the lowest number of clusters meaning that the clusters in CND-RA are bigger, i.e. consist of a greater number of cells per cluster than in the other groups. This suggests a more limited T cell epitope repertoire in CND-RA. The sequence logo plot of a cluster with the highest mean frequency among CND-RA patients compared to other subgroups is shown in **Figure 2D**.

Public clonotypes are TCR-sequences shared between different individuals, while private sequences are unique for each individual. Such clonotypes could be public either because they are more readily generated during thymic maturation or because these clonotypes are essential for the disease pathogenesis. To find the clonotypes that are important in disease pathogenesis, we resampled the groups to have the same number of samples (n=6 for each) and calculated the number of public clonotypes that are enriched to a given patient group. A low amount of enriched public clonotypes in a certain group would signify high similarity between the tested groups, while a high amount of enriched public clonotypes within a particular patient group would mean dissimilarity between the different tested groups. When SN-RA patients were compared to CND-RA, we found a median of 30 clonotypes that were enriched to SN-RA, and similarly, SP-RA patients had 12 such clonotypes, suggesting dissimilarity between the CND-RA and other RA subtypes (**Figure 2E**). When we pooled SP-RA and SN-RA patients together, we found more shared clonotypes than any other group combination (**Figure 2F**) again, suggesting that CND-RA is a distinct group from other RA groups.

### RA Patients Display Elevated Levels of Several Cytokines

**Figure 3:**
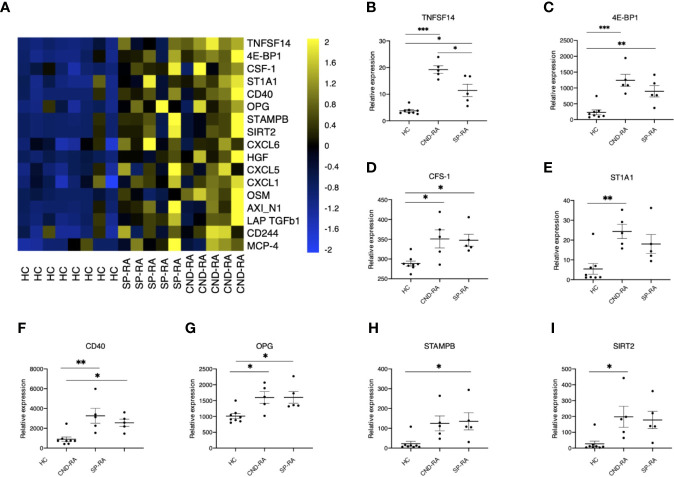
**(A)** Cytokine protein levels in healthy controls (HC), seropositive rheumatoid arthritis patients (SP-RA) and in ACPA-negative destructive rheumatoid arthritis (CND-RA) patients were measured using the Olink Pro seek inflammation panel. The heatmap displays all cytokines’ expression levels for which the Q-value (false discovery rate) was less than 0.035. Bright blue represents smaller protein concentration, while bright yellow represents higher protein concentration. **(B–I)** The expression levels of the top six differentially expressed cytokines expressed as NPX-units. Statistical testing with one-way ANOVA followed by Tukey post-hoc test. TNFSF14, tumor necrosis factor superfamily member 14; 4E-BP1, eukaryotic translation initiation factor 4E-binding protein 1; CSF-1, colony-stimulating factor 1; ST1A1, sulfotransferase 1A1; OPG, osteoprotegerin, tumor necrosis factor receptor superfamily member 11B; STAMPB, STAM binding protein; SIRT2, sirtuin 2. *p < 0.05, **p < 0.01, ***p < 0.001.

Cytokines are important mediators of inflammation, and their systemic levels can be used as a proxy to understand inflammatory mechanisms. Plasma samples were analyzed using a panel of 92 soluble inflammation mediators. The analyses revealed several upregulated cytokines in RA patient groups compared to healthy controls (**Figure 3A**). The only cytokine with significantly higher expression in CND-RA compared to SP-RA was the bone resorption associated cytokine TNFSF14 (LIGHT) (**Figure 3B**). There was no correlation between disease duration and cytokine levels in CND-RA. Other important inflammation regulators that were upregulated in RA (**Figures 3C–I****)** included CFS-1 (also known as M-CFS) (34) and CD40 that have been suggested as therapeutic targets in RA (35). In addition, osteoprotegerin (OPG) (36) levels were higher in RA groups than in healthy controls.

### Somatic Mutations Are Detected in an Expanded CD8+ Lymphocyte Clone

Somatic mutations are acquired changes in the cells’ genetic material and include single nucleotide variants (SNV), deletions and insertions as well as changes affecting larger parts of the genome. When the cell divides, also the mutations are passed to the following generations and after several rounds of cell division, the mutated cells form a clone. RA patients are known to harbor somatically mutated lymphocyte clones (16), while the biological significance of the observed mutations remain speculative. Modern deep sequencing technologies (eg. Exome-wide deep sequencing) have enabled identification of rare somatic variants with low, variant allele frequencies (VAF)s.

**Figure 4:**
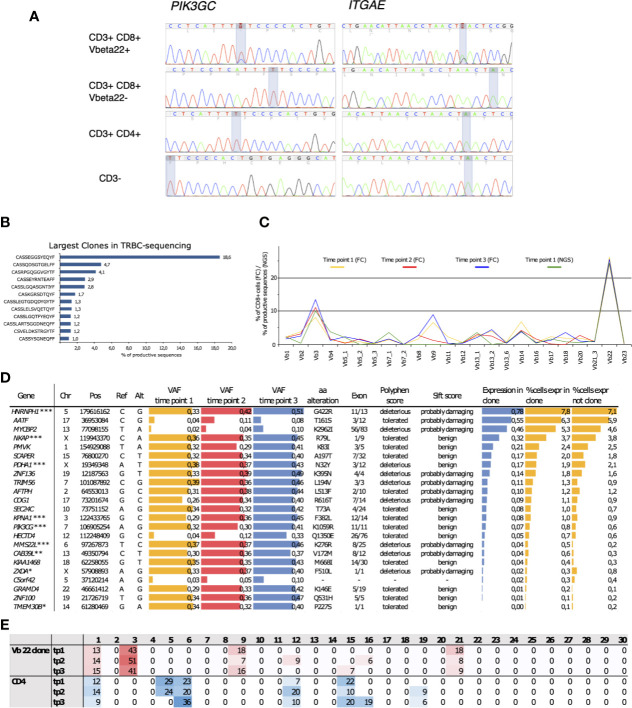
**(A)** Capillary sequencing was performed from flow cytometry sorted MNC fractions to confirm the immunopanel detected mutations and to confirm that they reside within the clonally expanded cell population. **(B)** The clonal expansion was confirmed to consist of an 18.6% monoclonal CD8+ population using NGS TCRB sequencing with the corresponding TCRBV02-01*01 beta-gene. **(C)** The clones were followed up at three time points spanning 4 years and 1 month using flow cytometry (FC). The sums of corresponding TCRBV genes were calculated from NGS TCRB sequencing. **(D)** The expanded clone harbored 23 somatic mutations that introduced an alteration into the mutated gene’s protein product and displayed an increasing variant allele frequency (VAF) over the follow-up period. Roughly half of the mutations were predicted as potentially deleterious for the protein function using the SIFT and PolyPhen prediction algorithms. The arbitrary expression levels of the mutated genes in the expanded clonal cells, as well as the percentages of cells expressing the mutated genes within the clonal cells and in cells outside the clone, were analyzed using sc-RNA-seq. *Lower gene expression levels in the clonal cells, **higher gene expression levels in the clonal cells, ***Somatic mutation confirmed by capillary sequencing. **(E)** Percentages of mutation signatures (1–30) in the flow-sorted CD3+CD8+ clonal cells compared to the polyclonal CD3+ CD4+ fraction. The most common signatures are highlighted with either red of blue shaded background. Chr, chromosome; Pos, position; Ref, reference base; Alt, mutated base; VAF, variant allele frequency; aa, amino acid; tp, time point.

An initial screen to identify somatic mutations in CD8+ cells was performed using the previously described gene panel (16). Only one patient out of the four tested carried somatic mutations. These SNVs were in *PIK3CG* and *ITGAE* genes, and they were restricted to the clonally expanded CD3+CD8+ cells (**Figure 4A**). TCRβ sequencing confirmed the presence of a *TRBV2* expressing clone matching with the observed VAFs and the Vbeta22 population seen in flow cytometry analysis (**Figure 4B**). Notably, the clonal landscape remained stable in all three analyzed time points spanning a follow-up period of over four years (**Figure 4C**).

**Figure 5:**
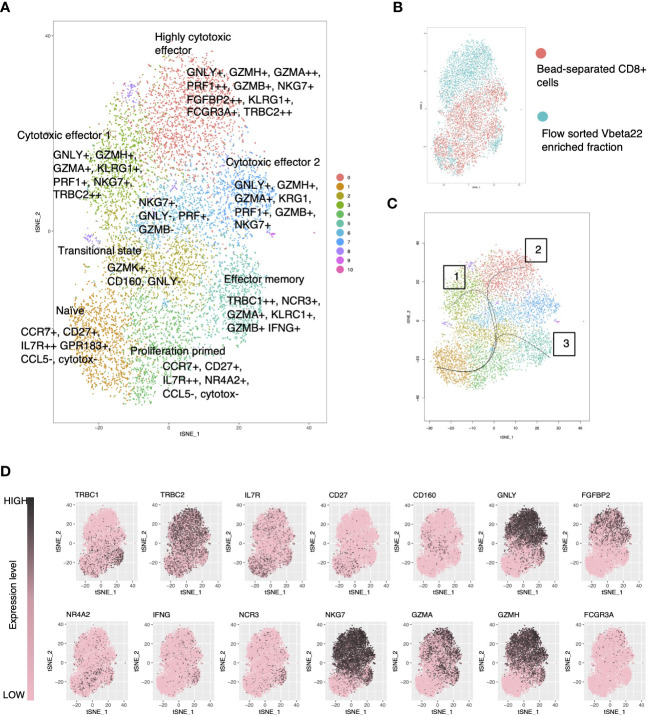
Single-cell RNA-sequencing reveals a cytotoxic gene expression signature in the clonally expanded, mutation harboring lymphocyte clone. **(A)** tSNE visualization of merged CD8+ cells with flow cytometry enriched clonally expanded CD8+ lymphocytes using Seurat-package. **(B)** Original identity of the cells. **(C)** Cell lineages using the naïve cells as the starting cluster were identified using the Slingshot tool. **(D)** Expression levels of selected genes over the clusters in tSNE visualization.

Whole-exome sequencing from the sorted Vb22 enriched cells followed by variant calling revealed 23 CNVs with increasing VAFs over the follow-up (**Figure 4D****)**. (The list of all identified somatic variants is presented in **Supplementary Table 3**.) By using in silico prediction tools and assessing the expression levels of the mutated genes, we aimed to identify gene alterations that could lead to phenotype level changes. Depending on the prediction algorithm, roughly half of these were likely to affect protein function. Gene expression levels within the clonally expanded, and in the polyclonal CD8+ cells, were retrieved from single-cell RNA sequencing (scRNA-seq) data (**Figure 5**). *MMS22L* and *CAB39L* displayed significantly higher expression levels within the clonal cells, while *ZXDA* and *TMEM30B* were less expressed within the mutation harboring cells. Six additional mutations were confirmed as restricted to the Vb22 expressing population by capillary sequencing (**Supplementary Figure 8****)**.

### Somatic Mutations in the Clonal Population Display a Unique Mutation Signature

Somatic mutations were called from the clonal CD8+ cells using the patient’s own CD4+ cells as germline reference. Thus, by reversing the analysis, we also identified variants that were specific for the CD4+ compartment. Mutation signatures are characteristic combinations of different types of mutations and they are widely used in cancer genomics. Mutation signature analysis takes into account the nucleotide change (from which nucleotide to which) and the genetic context (which are the adjacent nucleotides). Different mutational signatures are known to arise from different mutagenic processes such as exposure to genotoxic agents and mismatch repair deficiency (37). Analysis of mutational signatures (38, 39), revealed a predominant, and stable signature 3 in the clonal cells (**Figure 4E**), while the mutations within the CD4+ compartment were distributed to signatures 2, 6 and 15. There are no previous reports linking the signature 3 to T cell leukemias (40), while the particular signature has been associated with breast, ovarian and pancreatic cancers and with double-strand break-repair by homologous recombination.

### Single-Cell Level RNA-Sequencing Reveals a Cytotoxic Gene Expression Signature in the Mutation Harboring Lymphocyte Clone

ScRNA-seq data from the index patient’s CD8+ cells and flow-sorted Vb22 expressing cells were merged and clustered with the Seurat (29) toolkit using both PCA (**Figures 5A–D**) and CCA (**Supplementary Figures 2** and **3**) based methods. Vb22 sorted clonal cells (**Figure 5B**) accumulated to cluster 0 and 4, the first being a highly cytotoxic cluster with effector functions relying on both cytotoxic granule products (*GNLY* and *GZMA*) and overexpression of cytotoxicity associated receptors (*FGFBP2* and *FCGR3A)*. Lineage analysis identified three biologically relevant trajectories (**Figure 5C**). Lineages 1 and 2 described activation pathways for the clonal cells, while lineage 3 lead to a final phenotype of interferon-gamma producing cytotoxic effector cells more abundant in the non-clonal pool. The sorted Vb22 cells lacked *TRBC1* expression and displayed an increased proportion of *TRBC2* (**Figure 5D**) expressing cells, which further confirmed that the clonal cells resided in the clusters 0 and 4.

## Discussion

Recently, we described a distinct SN-RA cohort with the destruction of larger joints (3). Here we sought to understand the biological mechanisms of this subtype of RA.

In addition to the diagnostic antibodies, RA patients are also known to develop antibodies against other endogenous proteins and post-translational modifications (30, 41–44). Our findings show that CND-RA patients display fewer antibodies against endogenous proteins than SP-RA patients. The lower number of autoantibodies in CND-RA supports the idea of this RA subtype being an autoantibody independent disease subtype. A similar, but an earlier version of the Protoarray platform has previously been used to examine autoreactive antibodies in HLA-restricted RA, other rheumatic diseases and healthy controls (43). In their work, Auger et al. identified significantly more autoreactive antibodies in all these conditions. This discrepancy to our findings likely originates from the different strategy in setting the positivity threshold. Probably due to our small cohort size, we could not reproduce the RA-associated positive autoreactivities against PAD4, BRAF, PKCβ1or PIP4K2C detected by Auger et al.

Further, CND-RA patients’ plasma samples were found to display higher concentration of TNFSF14 (also known as LIGHT), a cytokine known to mediate bone resorption in RA, also matching well with the destructive erosive phenotype (45). Notably, CND-RA patients with ongoing immunosuppressive treatment also displayed high levels of other pro-inflammatory cytokines. We interpret this as an indication of ongoing, treatment resistant inflammation.

Each T-cell recombines its T-cell receptor (TCR) during thymic maturation leading to a situation where each naïve T-cell has a unique TCR sequence. Upon contact with its cognate antigen, it proliferates and converts into an effector or effector memory lymphocyte. Sequencing of an individual’s TCRβ repertoire can be used to reveal past infections such as CMV, Epstein-Barr virus and influenza (26), while also autoimmune diseases such as type 1 diabetes (46), ankylosing spondylitis (47, 48) and psoriasis (49) have been suggested to associate with disease-specific TCR signatures. The majority of the reported TCR studies in RA have focused on CD4+ T-helper cells (50, 51).

We have previously studied the CD8+ TCRβ lymphocyte repertoires in early RA patients (16, 52), and here we focused on the CD8+ TCRβ repertoires in CND-RA patients. We identified a less diverse TCR-repertoire in CND-RA than in healthy controls, SP-RA or in SN-RA. Thus, our data showed that the CD8+ lymphocyte compartment in CND-RA consisted of fewer but larger clones than in other RA-subtypes. CND-RA patients also displayed a pronounced CMV-associated TCRβ-repertoire balanced by a significantly reduced influenza A-associated TCRβ signature. Interestingly, CMV seropositivity has previously been linked to more severe joint destruction in RA (53). Importantly, CND-RA patients had a unique set of enriched public clonotypes, further supporting our hypothesis of CND-RA being a separate disease from diagnostic SP-RA and SN-RA. However, with the present data set we cannot exclude the possibility that long-term inflammation with continuous attempts to pharmaceutically suppress it, may be reflected in the patients’ TCR repertoires.

Clonally expanded CD8+ effector T cells have previously been shown to harbor somatic mutations that potentially modulate their phenotype and effector functions (16, 19, 20). Knowing that CND-RA differs from SP-RA in terms of CD8+ pool composition, we studied whether these patients harbor somatic mutations in their CD8+ cells. Our data further confirm the recurring presence of somatic mutations in expanded effector clones, but as the findings are from a single patient, somatic mutations cannot be considered as part of the characteristic CND-RA signature.

We hypothesized that the biologically most important mutations are likely to enrich in the (sub)clonally expanding cells and thus, we focused on mutations in expressed genes with growing VAFs in the follow-up samples. We identified 23 coding single nucleotide variants that expanded in size over time. Interestingly, the mutational signature within the mutated clone was stable during the follow-up and differed from that observed in the non-clonal cells. These cytotoxic T cells’ mutational landscape did not follow the mutational patterns previously observed in T-cell leukemias (T-LGL and T-PLL) (40). Somatic mutations are hallmarks of cancer. However, somatic mutations are also found in morphologically normal tissue (11, 13). In our work, the identified somatic mutations are restricted to mature lymphocyte clones, and thus, they are unlikely to associate with the development of a malignant disease. The mutation harboring CD8+ T cell clone was found to express high levels of cytotoxic gene products and molecules associated with pro-inflammatory signaling. This highlights the possibility that the mutated clone may be involved in the propagation of chronic inflammation in the index patient.

The limitations of the study include the size of the CND-RA cohort. Considering the rarity of the observed clinical phenotype and the time needed today to establish the diagnosis of CND-RA, we are confident that the cohort size is sufficient to define this RA subset as an independent disease subclass. However, without sample material from the diagnostic time point, we cannot conclusively state whether the observed differences in TCR-repertoires compared to SP- and SN-RA are present at onset or whether they are, at least in part, due to poor disease control over the years.

In conclusion, we have identified a specific TCR signature from the CD8+ cytotoxic T cells from CND-RA patients. While the pathogenesis of SP-RA is driven by MHC II and CD4+ T helper lymphocytes, the observed CD8+ T cell signature may be linked to the pathogenesis of CND-RA. Narrower autoreactive antibody repertoire also fits well with the observed CD8+ TCR repertoire phenotype as antibody production is dependent on T helper cells, not on CD8+ cells. As the conventional antirheumatic compounds do not halt disease progression in CND-RA, we hypothesize that these patients would potentially benefit from treatment strategies that target immune signaling that is essential for CD8+ cells, e.g. compounds that inhibit JAK-STAT signaling. Our results emphasize the importance of understanding the various immunopathological mechanisms operating in the different subtypes of RA, so that future research can extend this knowledge to yield improved therapeutic options for the treatment resistant patients.

## Data Availability Statement

TCRβ sequencing data is freely available at clients.adaptivebiotech.com/pub/kelkka-2020-jci (DOI: 10.21417/TK2020FI). All other datasets presented in this study are included in the article/**Supplementary Material**.

## Ethics Statement

The studies involving human participants were reviewed and approved by Helsinki University Hospital Ethics Committee. The patients/participants provided their written informed consent to participate in this study.

## Author Contributions

All authors were involved in drafting the article or revising it critically for important intellectual content, and all authors approved the final version to be published. SM and TK had full access to all of the data in the study and take responsibility for the integrity of the data and the accuracy of the data analysis. Study conception and design: TK, PS, TS-I, and SM. Acquisition of data: TK, PS, TL, KP, MT, ML, PE, RK, CS, KS, YM, HN, ML-R, and TS-I. Analysis and interpretation of data: TK, PS, DB, JH, TL, MK, JS, SE, BY, SK, LE, HL, TS-T, and SM. All authors contributed to the article and approved the submitted version.

## Funding

European Research Council (Projects: M-IMM 647355, STRATIFY 862011, DynaOmics 677943), Academy of Finland Heal-Art consortium (314442) and ERA PerMed (JAKSTAT-TARGET consortium), Maire Lisko foundation, Finnish Medical foundation, Instrumentarium Science foundation, Biomedicum Helsinki foundation, Orion research foundation, Juhani Aho foundation, K. Albin Johansson foundation, Paulo foundation, Region Östergötland (ALF grants), European Union’s Horizon 2020 research and innovation programme (675395), Tekes the Finnish Funding Agency for Innovation (1877/31/2016), Sigrid Juselius Foundation, Finska Läkaresällskapet, and Liv och Hälsa Foundation.

## Conflict of Interest

The authors declare that the research was conducted in the absence of any commercial or financial relationships that could be construed as a potential conflict of interest.
