# Corrigendum: Adult-Onset Anti-Citrullinated Peptide Antibody-Negative Destructive Rheumatoid Arthritis Is Characterized by a Disease-Specific CD8+ T Lymphocyte Signature

**DOI:** 10.3389/fimmu.2021.710831

**Published:** 2021-05-31

**Authors:** Tiina Kelkka, Paula Savola, Dipabarna Bhattacharya, Jani Huuhtanen, Tapio Lönnberg, Matti Kankainen, Kirsi Paalanen, Mikko Tyster, Maija Lepistö, Pekka Ellonen, Johannes Smolander, Samuli Eldfors, Bhagwan Yadav, Sofia Khan, Riitta Koivuniemi, Christopher Sjöwall, Laura L. Elo, Harri Lähdesmäki, Yuka Maeda, Hiroyoshi Nishikawa, Marjatta Leirisalo-Repo, Tuulikki Sokka-Isler, Satu Mustjoki

**Affiliations:** ^1^ Hematology Research Unit Helsinki, University of Helsinki, Helsinki, Finland; ^2^ Department of Hematology, Helsinki University Hospital Comprehensive Cancer Center, Helsinki, Finland; ^3^ Department of Clinical Chemistry and Hematology, University of Helsinki, Helsinki, Finland; ^4^ Translational Immunology Research Program, University of Helsinki, Helsinki, Finland; ^5^ Turku Bioscience Centre, University of Turku and Åbo Akademi University, Turku, Finland; ^6^ Rheumatology, Jyväskylä Central Hospital, Jyväskylä, Finland; ^7^ Institute for Molecular Medicine Finland (FIMM), Helsinki Institute of Life Science (HiLIFE), University of Helsinki, Helsinki, Finland; ^8^ Rheumatology, University of Helsinki, Helsinki University Hospital, Helsinki, Finland; ^9^ Department of Biomedical and Clinical Sciences, Division of Inflammation and Infection, Linköping University, Linköping, Sweden; ^10^ Institute of Biomedicine, University of Turku, Turku, Finland; ^11^ Department of Computer Science, Aalto University School of Science, Espoo, Finland; ^12^ Division of Cancer Immunology, Research Institute/Exploratory Oncology Research and Clinical Trial Center (EPOC), National Cancer Center, Tokyo, Japan; ^13^ University of Eastern Finland, Faculty of Health Sciences, Kuopio, Finland

**Keywords:** rheumatoid arthritis, seronegative, ACPA-negative, CD8+ lymphocyte, T cell receptor, somatic mutation

An author name was incorrectly spelled as **Hiroyashi Nishikawa**. The correct spelling is **Hiroyoshi Nishikawa**.

The authors apologize for this error and state that this does not change the scientific conclusions of the article in any way. The original article has been updated.

