# Supplementary material for: Adult-Onset Anti-Citrullinated Peptide Antibody-Negative Destructive Rheumatoid Arthritis Is Characterized by a Disease-Specific CD8+ T Lymphocyte Signature
Source: Front Immunol. 2020 Nov 19;11:578848. doi: 10.3389/fimmu.2020.578848 (PMC7732449; doi:10.3389/fimmu.2020.578848)
Supplement: Supplementary file 1 [file DataSheet_1.pdf]

Table of Contents 1

Supplementary Materials and Methods 2

Supplementary Figures

Supplementary Figure 1. Flow cytometry sorting and purities for sc-RNA-seq 5
Supplementary Figure 2. Origin of cells in CCA clustering of sc-RNA-seq data 6
Supplementary Figure 3. CCA clustering of the sc-RNA-seq data 7
Supplementary Figure 4. Autoantibodies 8
Supplementary Figure 5. Largest clones in CD4+ and CD8+ lymphocytes 9
Supplementary Figure 6. TCR-repertoire correlations 10
Supplementary Figure 7. Virus specific sequences 11
Supplementary Figure 8. Somatic mutations in capillary sequencing 12

Supplementary Tables

Supplementary table 1. Extended clinical data 13
Supplementary table 2. Patient characteristics for SP-RA and SN-RA 14
Supplementary table 3. Complete list of variants 15

Supplementary materials and methods

Isolation of CD4+ and CD8+ cells

CD4+ and CD8+ cells were separated either from fresh or from cryopreserved (10 % DMSO in FBS) PBMCs with magnetic bead sorting using positive selection for both fractions (AutoMACS, Miltenyi Biotec). Purities of the sorted fractions were confirmed with flow cytometry (FACSAria II, Becton Dickinson).

DNA isolation

DNA was extracted from CD4+ and CD8+-samples or from the MNC fraction with Nucleospin Tissue DNA extraction kit (Machery Nagel) or Nucleospin Tissue XS kit (Machery Nagel) according to the manufacturer's instructions. DNA concentration was measured with Qubit 2.0 fluorometer (Life Technologies).

Autoantibody screen and analysis

Autoantibodies were screened from plasma samples using the Invitrogen (Carlsbad, California, USA) ProtoArray protein microarray microarray v.5.1 (https://www.thermofisher.com/jp/en/home/life-science/protein-biology/protein-assays-analysis/protein-microarrays.html) as previously described in [1]. All proteins have been expressed as glutathione-S-transferase (GST) fusion proteins, purified under native conditions and spotted on nitrocellulose-coated glass slides. Slides were blocked with 1% BSA/phosphate-buffered saline/Tween (PBST). Plasma samples were added to the arrays. After washing, anti-human immunoglobulin G (IgG) conjugated to Alexa Fluor 647 dye was added. Arrays were washed and dried (Partnership, Evry, France). Arrays were scanned with a GenePix 4000B Fluorescent Scanner, GenePix.Molecular Devices, Sunnyvale, California, USA. Data were acquired with GenePix Pro software and processed using ProtoArray Prospector 2.0 (Invitrogen). A panel of values was calculated for each protein array, including the Z-score, the Chebyshev inequality precision (CIP) value and the coefficient of variation (CV) value as previously described [3]. A Z-score >3.0, a CIP value <0.05 and a CV <0.5 define a positive spot.

Cut-off for sample positivity was set to FC10 (calculations) and to FC 15 (illustrations) compared to the average of healthy controls. Assay performance was confirmed by a Europium labeled sandwich DELFIA against therapeutic anti-TNFa antibodies present in prediluted (1:30) patient samples. Briefly, 96-well plates were coated with recombinant TNFa (Novus biologicals, NBC1-18460) and bound antibodies were detected using Europium labeled anti-human-IgG (Perkin Elmer, 1244-330) and Victor plate reader (Perkin Elmer).

Immunogene panel sequencing

A probe based panel of 986 genes was used to discover somatic mutations in immune related genes as described in [2]. Reads were aligned to the reference genome GRCh37 following variant idenfication using VarScan2 based bioinformatic pipeline for mutation detection that is described in [2]. Variant calling was performed both using CD4+ cells and CD8+ cells as germline.

KAPA Hyper Library preparation and SeqCap EZ Enrichment

150 ng of dsDNA was fragmented with Covaris E220 evolution instrument (Covaris, Woburn, MA, USA) to mean fragment size of 200 bps. Sample libraries and enrichment were processed according to SeqCap EZ HyperCap Workflow User's Guide (Roche Nimblegen, Madison, WI, USA) using KAPA Hyper library preparation kit with the following modifications. 2 µl of 15 µM xGen® Dual Index UMI Adapters (Integrated DNA z, San Jose, CA, USA) was used in ligation. Four samples were pooled to enrichment, using 1 µg of each library. SeqCap EZ MedExome probes (Roche NimbleGen) were used in the enrichment. The amplified library was purified with 1.8x Agencourt

AMPure XP beads and eluted to 200µl EB buffer. Library was quantified for sequencing using LabChip High Sensitivity Assay (PerkinElmer, Waltham, MA, USA) and Kapa Library Quantification Kit Illumina (Kapa Biosystems, Wilmington, MA, USA).

Sequencing was performed with the Illumina HiSeq2500 system in HiSeq high output mode using v4 kits (Illumina, San Diego, CA, USA). Read length for the paired-end run was 2x101 bp. Sequencing was performed in FIMM Technology center.

Variant identification
Somatic variants were identified with the GATK toolkit and MuTect2 as previously described in [3]. To discover variants occurring only in CD4+ or CD8+ cells, variant calling was performed as a paired-sample analysis, using CD4+ cells as a germline control and CD8+ as a "tumor" sample, and vice versa. This procedure was used to exclude germline-derived variants.

Sequencing data was pre-processed with the Trimmomatic software [4], and aligned to the genome with BWA-MEM (arXiv:1303.3997). Base recalibration was performed with GATK BaseRecalibrator [5]. GATK IndelRealigner was used for local realignment near indels. The Picard toolset was used to filter PCR duplicates. ContEst was used to estimate cross-sample contaminations.

All variants were required to pass all MuTect2 filters. GATK3 tools were used, with the exception that 8-oxoguanine and deamination artifacts and possible cross-sample contaminations were estimated and filtered by GATK4 CalculateContamination, CollectSequencingArtifactMetrics, and FilterByOrientationBias. Reads with quality <40 quality and with coverage <10 were filtered out. In addition, variants with strand orientation bias (SOR)of >3 for single-nucleotide alterations and >11 for indels were excluded.

Variants were annotated with Annovar [6]. Variants with a population frequency >1% in gnomAD_exome_ALL, gnomAD_exome_FIN, gnomAD_exome_NFE, esp6500siv_2_all, or 1000g2015aug_all were filtered.

All reported variants were identified with at least 7 supporting reads in all 3 timepoints. In addition, read counts from all time points for variant loci present in less than 3 timepoints are also reported. All reported variants were manually validated using IGV.

Capillary sequencing
DNA from the flow-sorted cell fractions was subjected to 30 PCR cycles with primers (10 uM) and annealing temperatures indicated in **Methods table 1**. Phusion High-Fidelity DNA Polymerase (Thermo Scientific, #F530L) was used according to the manufacturer's instructions. The PCR products were cleaned using Clean Sweep (Thermo, #A29896).

The amplified PCR product was mixed with 8 pmol sequencing primer and subjected to BigDye v.3.1 sequencing with dye terminator removal with Optima DTR on 96-Well filter plates. Electrophoresis was carried out with ABI3730xl DNA Analyzer and base calling was performed with Sequencing Analysis 5.2 followed by sequence visualization by 4Peaks software. Sequencing was performed in FIMM Technology center.

Methods table 1. Primers and PCR conditions for capillary sequencing.

Gene	Amino Acid	Position	nt	fwd primer	rev primer	Tm (°C)	Length (bp)
HNRNPH1	G422R	5:179043163	C < G	CAAACCAGTCCAGCTACGGG	ACCTGCCTGCGACTTACTCT	65	210
ITGAE	N860D	17:3638188	T > C	GTCCTATTGTATTGCGTTGAGGTTT	GGACTGGCGGGGATTTATCT	60	361
KPNA1	F382L	3:122152612	G > C	ACAGGAGAAGGGGTGTTAATAGT	TTCCCCTCAAAGGCAGCTAAT	60	353
MMS22L	K276R	6:97715749	T > C	CTGGCAAGTGACAATTTAACCAA	TTAGGCTTCCAGTGATTTCGGT	60	404
NKAP	R79L	X:119077333	C > A	AGTCCGAAGCCCAGCAAATC	AGATCGGACTCCAAAGGCAC	65	367
PDHA1	N70Y	X:19367466	A > T	CTTGCCTTCAGATGCGGGAT	ACAGAACAACAGCAAGCTGCTAA	60	373
PIK3CG	K1059R	7:106545699	A > G	GATGACAGGAATGCCCCAGT	CCAAACAATCAGCAATGCCA	65	384

sc-RNA-seq

Canonical Correlation Analysis (CCA) using the Seurat toolkit was performed between the two single cell datasets to identify common sources of variations and the datasets were aligned using top 1200 highly variable genes from each dataset (252 overlapping highly variable genes between two datasets). Top 25 CC's were used for downstream clustering and 5 different clusters were identified (**Supplementary Figure 2**).

For the CCA based analysis, 333 cells were found to be specific for the flow-cytometry enriched Vbeta22 expressing clonal cells dataset and were thus removed from the analysis (as they can be regarded as dataset specific cells). These cells were retraced to the PCA based clustering plotted on tsne based visualization (**Supplementary Figure 3**). Most cells were seen in the biggest cluster, cluster 0. To further understand why these cells were specific to the flow-cytometry enriched Vbeta22 expressing cells, this data was analyzed separately with PCA dimensionality reduction, clustering and followed by tSNE visualization. These 333 cells when plotted on the tSNE clustering, were seen scattered over multiple clusters which rules out the possibility of these cells being a rare cell population.

Gene expression analysis

mRNA expression data for the healthy controls has previously been reported in [2]. Gene expression data as well as differentially expressed genes in the expanded clone were retrieved from the time point 1 clonal cells that were analyzed with the 10x single cell mRNA-seq assay.

Supplementary references

1 Hamano Y, Kida H, Ihara S, *et al.* Classification of idiopathic interstitial pneumonias using anti–myxovirus resistance-protein 1 autoantibody. *Sci Rep* 2017;**7**:1–15. doi:10.1038/srep43201

2 Savola P, Kelkka T, Rajala HL, *et al.* Somatic mutations in clonally expanded cytotoxic T lymphocytes in patients with newly diagnosed rheumatoid arthritis. *Nature Communications* 2017;**8**:15869.

3 Savola P, Martelius T, Kankainen M, *et al.* Somatic mutations and T-cell clonality in patients with immunodeficiency. *Haematologica* 2019;**Online ahead of print**. doi:10.3324/haematol.2019.220889

4 Bolger AM, Lohse M, Usadel B. Trimmomatic: a flexible trimmer for Illumina sequence data. *Bioinformatics (Oxford, England)* 2014;**30**:2114–2120.

5 McKenna A, Hanna M, Banks E, *et al.* The Genome Analysis Toolkit: a MapReduce framework for analyzing next-generation DNA sequencing data. *Genome research* 2010;**20**:1297–1303.

6 Wang K, Li M, Hakonarson H. ANNOVAR: functional annotation of genetic variants from high-throughput sequencing data. *Nucleic Acids Research* 2010;**38**:e164–e164.

Supplementary Figure 1. Flow cytometry sorting and purities for sc-RNA-seq

A

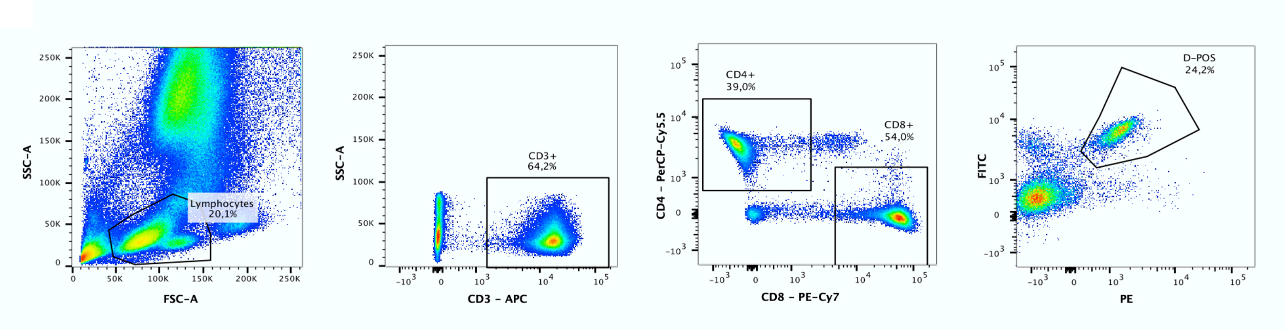

B

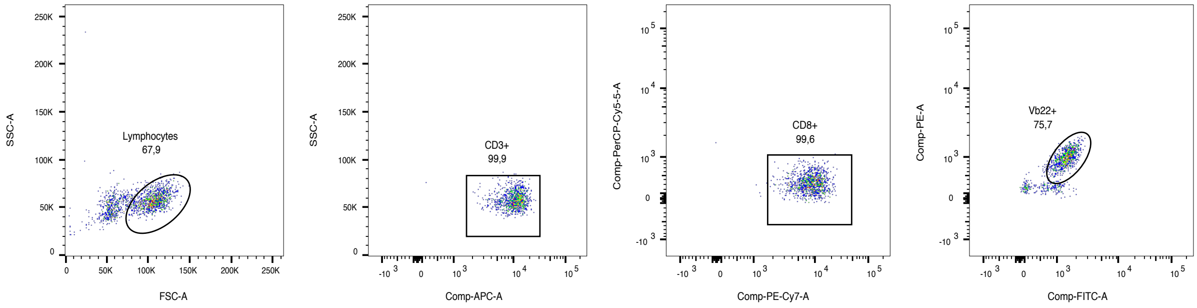

C

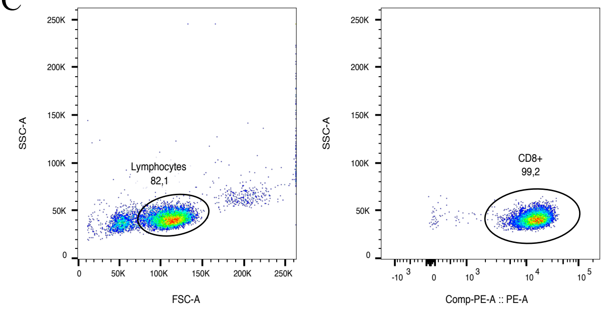

Supplementary Figure 1. A: Gating strategy to define the clonally expanded CD3+ CD8+ lymphocytes in the peripheral blood of the index patient. Vb22 binding antibodies were labeled with both FITC and PE and the expanded clone (24%) is displayed as double positive in the far-right panel. **B**: Cells in sc-RNA-seq analyses. Gating strategy and purity analysis for Vb22 enriched CD3+CD8+ lymphocytes that were loaded in 10x-controller. **C:** Purity of the bead separated CD8+ cells loaded in the 10x-controller.

Supplementary Figure 2. CCA clustering of the sc-RNA-seq data

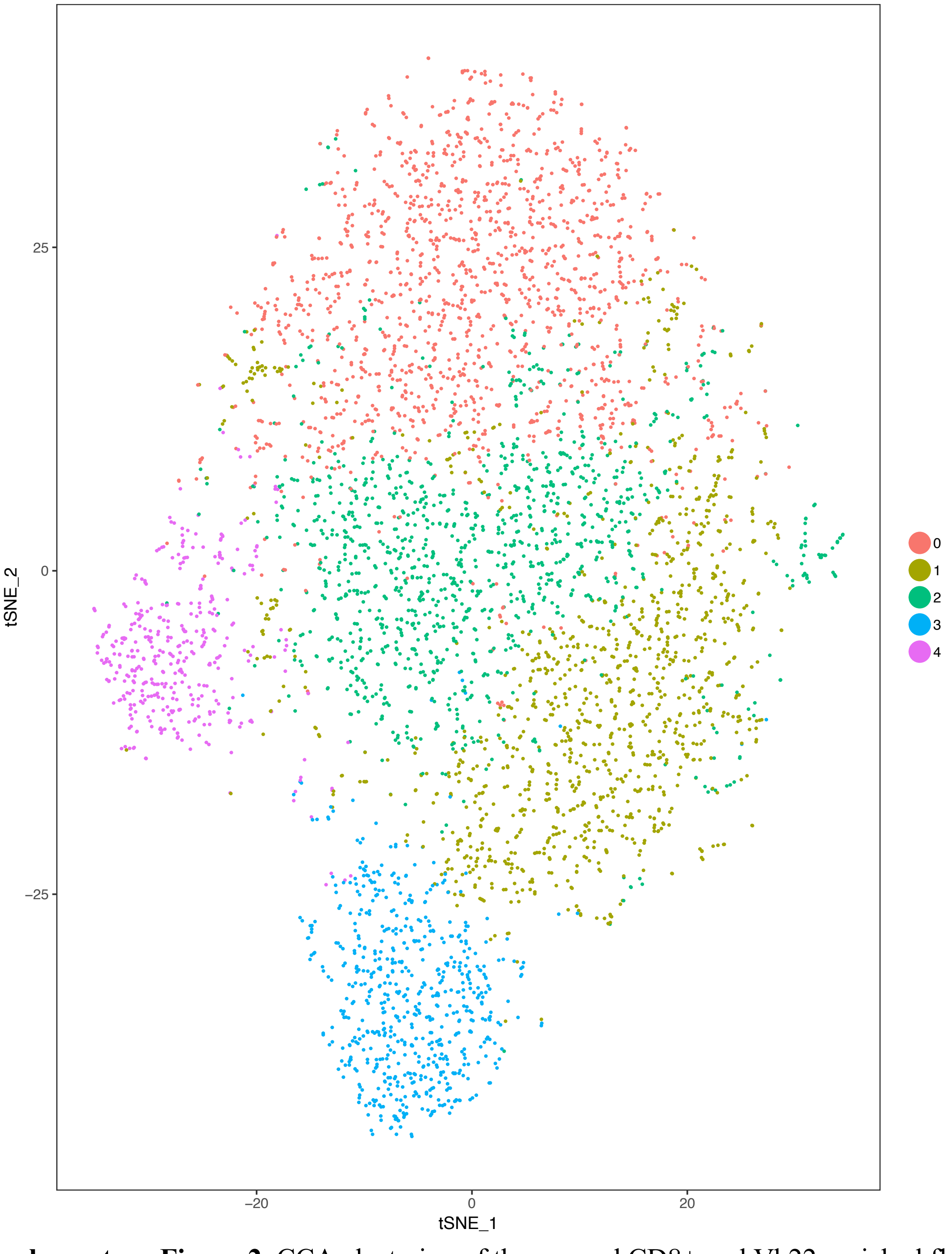

Supplementary Figure 2. CCA clustering of the merged CD8+ and Vb22 enriched flow-sorted cells divided the cells into five main clusters.

Supplementary Figure 3. Origin of cells in CCA clustering of sc-RNA-seq data

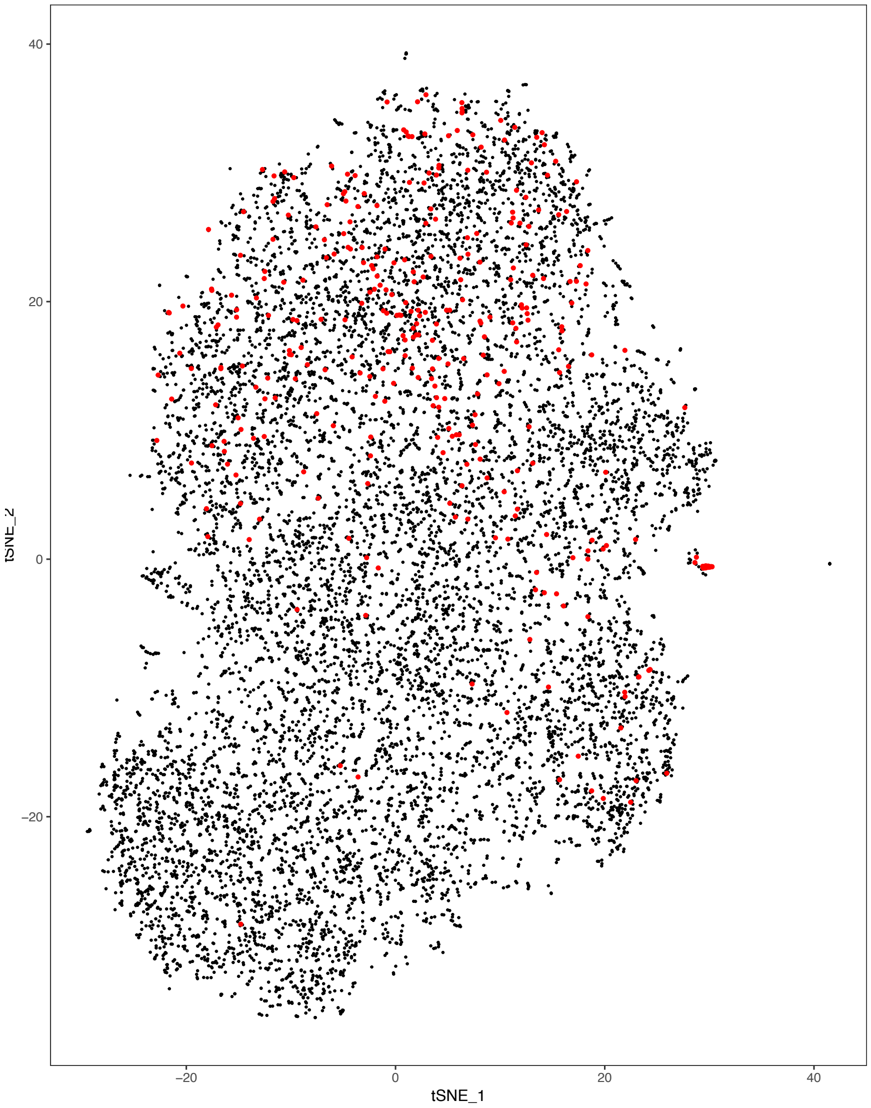

Supplementary Figure 3. CCA clustering of the CD8+ (black) and Vb22 enriched flow-sorted cells (red) reveals the same cell distribution as the PCA method.

Supplementary Figure 4. Autoantibodies

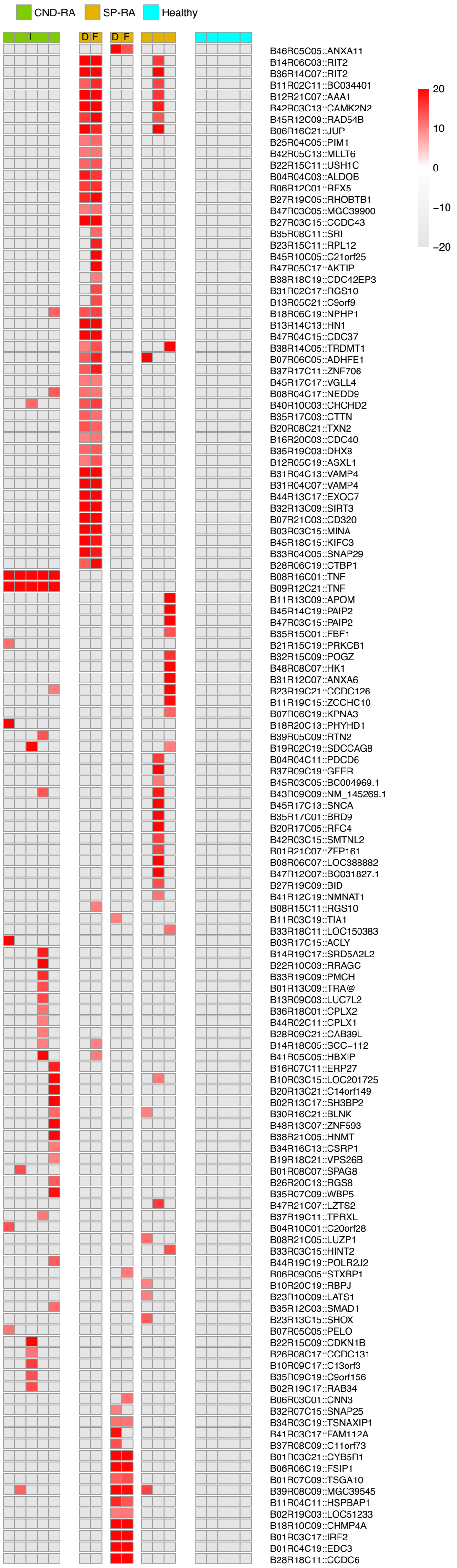

Supplementary Figure 4. Antibodies against endogenous proteins are reported as FC to the mean of healthy controls. All targets with FC > 15 are included in the Figure. D = sample taken at diagnosis, F = sample taken after a follow-up period of 14 or 16 months, I = index patient.

Supplementary Figure 5. Largest clones in CD4+ and CD8+ lymphocytes

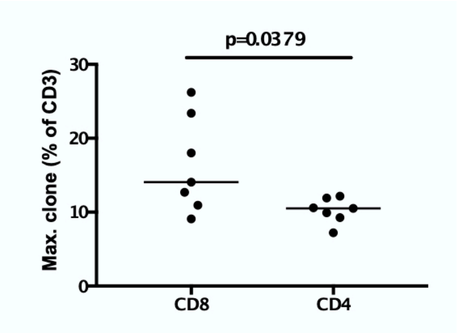

Supplementary Figure 5. The relative sizes of different v-beta (Vb) family expressing lymphocyte populations within each patients' CD4+ and CD8+ lymphocytes were compared using flow cytometry. The largest Vb restricted populations in both CD8+ and CD4+ lymphocytes were plotted. Bar indicates the median values, unpaired, two-tailed Man-Whitney.

Supplementary Figure 6. TCR-repertoire correlations

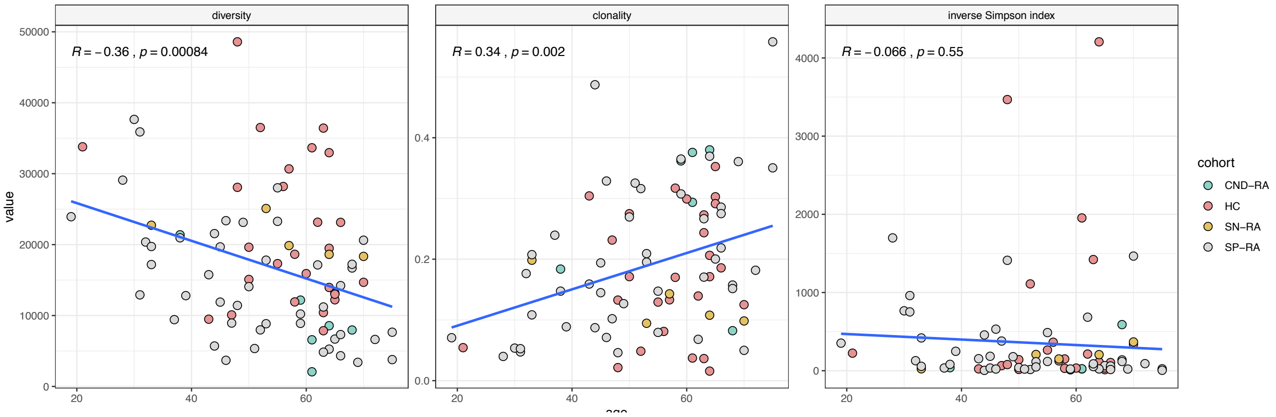

Supplementary Figure 6. Impact of age on sample diversity, clonality and Inverse Simpson index.

Supplementary Figure 7. Virus-specific sequences

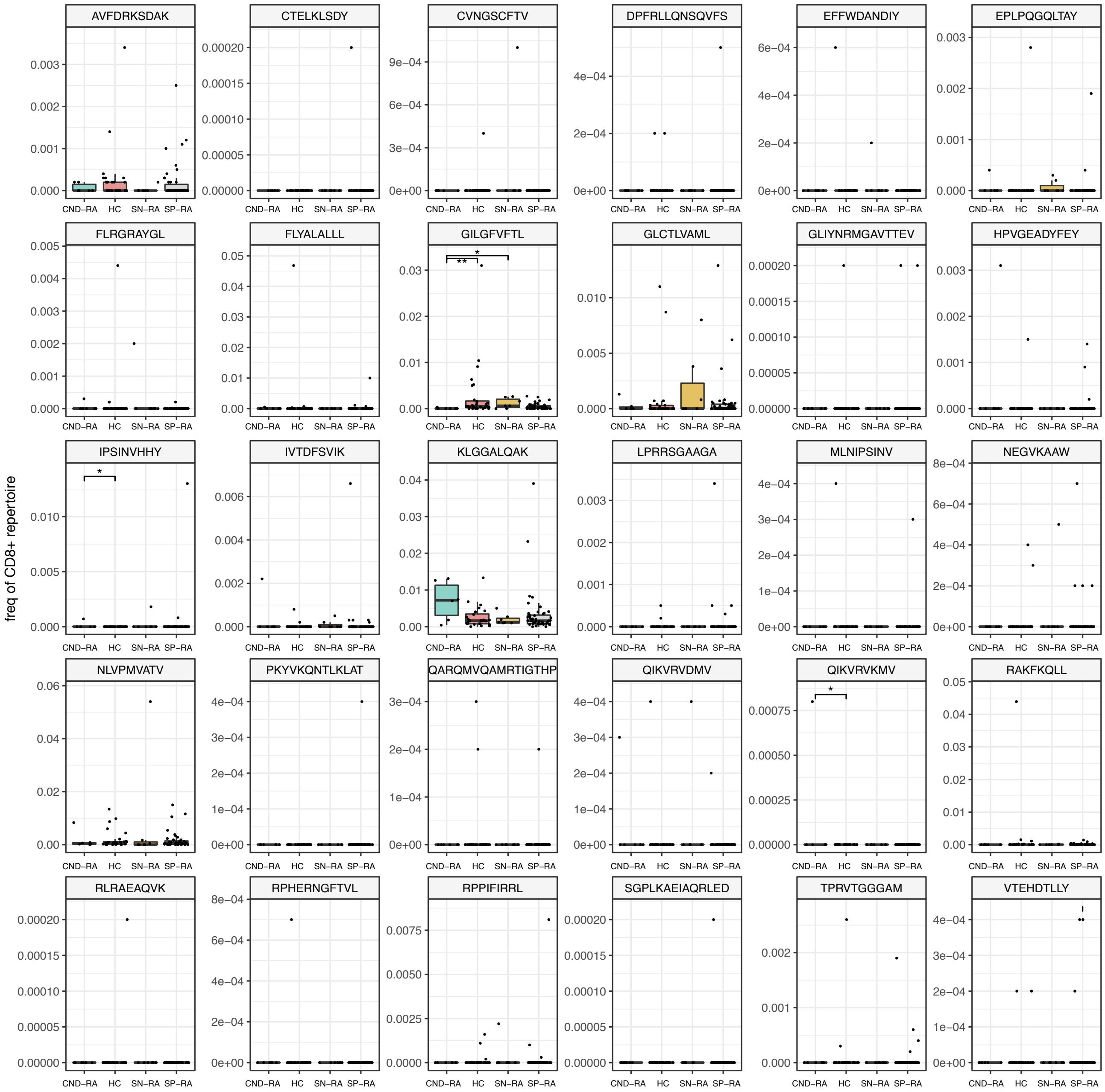

Supplementary Figure 7. Frequencies of known virus-associated TCR-sequences sorted by the immunodominant peptides.

Supplementary Figure 8. Somatic mutations in capillary sequencing

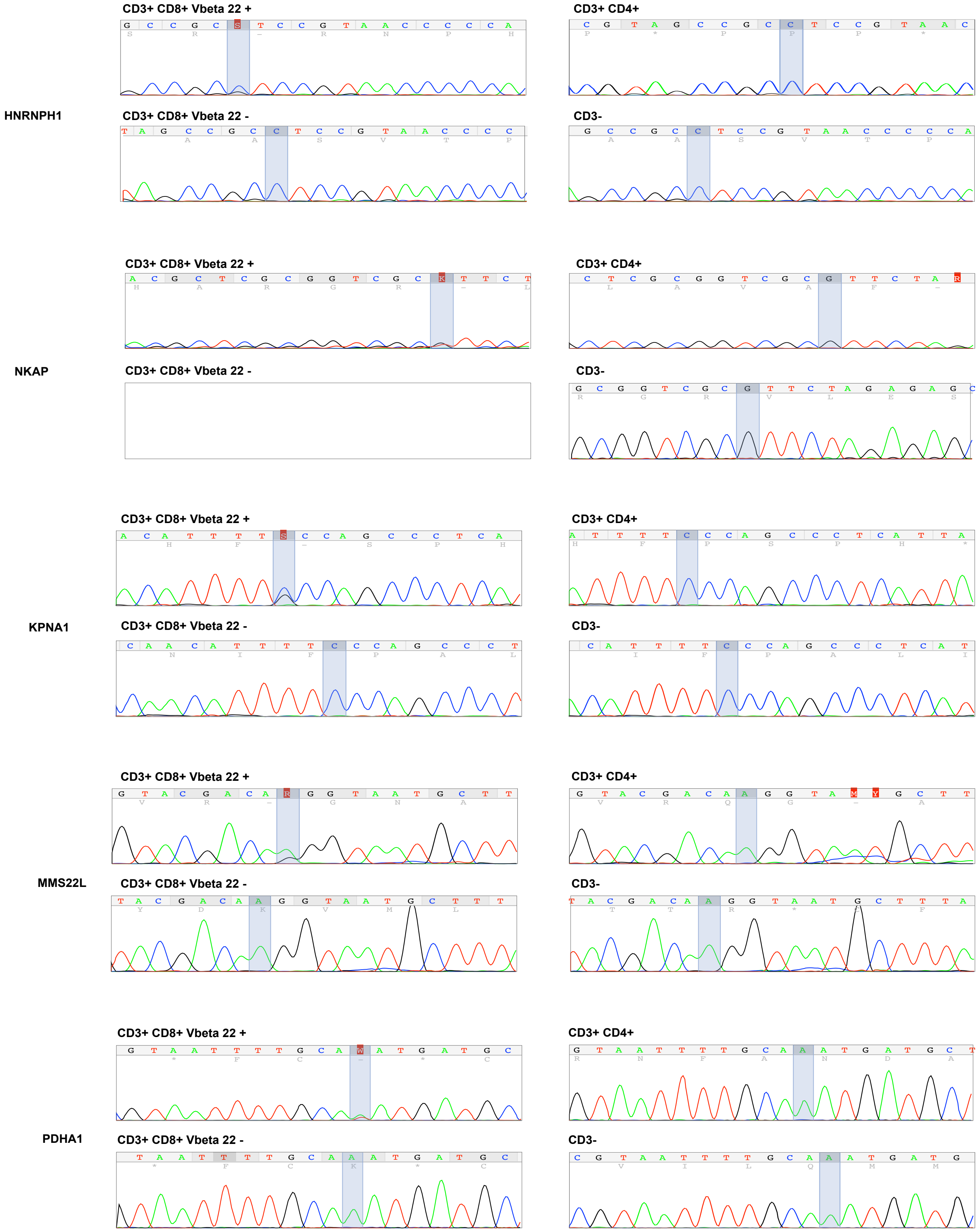

Supplementary Figure 8. Somatic mutations confirmed in capillary sequencing. Sequencing data for the NKAP gene in the Vbeta22- fraction is not available due to sample availability.
Somatic mutations in capillary sequencing

Supplementary Table 1. Extended clinical data. All data points were collected at the time of sampling.

Patient	Largest clone (% of CD8+)	Largest clone NGS (% of CD8+)	Age at dg	Age at sampling	Hb (g/l)	ESR (mm/h)	B-leuc (10^9	Lymph (%)	Mono (%)	Nf (%)	Baso (%)	Eos (%)	Plt (10^9)	CRP (mg/l)	RF (IU/ml)	ACPA (U/ml)	DAS28	tender joints (n)	swollen joints (n)	pat global	HAQ	aCarb	HLA-B27	treatment at sampling	non-responsive to
**Pt-1**	18	8,5	45-55	65-75	136	24	8,1	40	8,1	44	0,5	3,8	378	3	<30	<7	4,2	5	1	28	1,9	Neg	+	ADA, MTX	INF
**Pt-2**	9	4,9	25-35	55-65	126	23	8,2	27,2	9,9	48,3	0,9	9,9	486	3	<30	<7	2,5	1	1	16	1,8	Neg	+	ETA, ABA, MTX	INF
**Pt-3**	26	24,5	25-35	55-65	147	19	7,8	35,5	9	50,5	0,5	4,5	235	3	<30	<7	3,4	1	1	16	1,4	Neg	-	INF, MTX, PRED	CYA. HXC. ATT, LEF, SUL
**Pt-4**	13	16,3	15-25	35-45	129	5	7,6	20	3,4	74,4	0,3	1,1	370	0	<30	<7	1,4	1	1	31	0,3	Neg	-	ETA, MTX, PRED	RIT, INF
**Pt-5**	14	21,2	55-65	55-65	112	42	12,3	9,8	5,4	83,7	0,1	0,6	486	109	<30	<7	4,6	4	3	30	0,5	ND	-	INF, MTX, PRED	INF
**Pt-6**	23	16,3	45-55	55-65	116	8	5,7	37,8	9,1	32,6	1	16,7	251	1	<30	<7	1,7	0	0	67	2,1	ND	-	ETA, MTX, HXC	-
**Pt-7**	ND	1,4	45-55	65-75	126	8	4,9	18	5,5	72,3	0,9	1	261	1	32	8	1,7	0	0	53	2,0	ND	-	ABA. MTX, PRED	INF, SUL, HXC
**Pt-8**	ND	12,3	25-35	55-65	133	9	10,5	36	6,5	51,1	0,6	4,2	297	2	37	<7	2,7	3	1	26	2,6	ND	-	HXC, PRED	CYA, MTX, ATT,POD

ADA	Adalimumab
MTX	Metotrexate
INF	Infliximab
ETA	Etanercept
ABA	Abatasept
PRED	Prednisolon
CYA	Cyclosporin
HXC	Hydroxichloquin
ATT	Atsatioprin
LEF	Leflunomid
SUL	Sulfasalatzine
RIT	Rituximab
POD	Podofyllotoxin

Supplementary Table 2. Patient characteristics for SP-RA and SN-RA.

Patient ID	Gender	Age at diagnosis	ESR	CRP	RF	CCP-ab	SEROPOS
13	F	15-25	8	4	4	6	N
15	F	65-75	57	63	14	18	N
19	F	25-35	5	4	11	7	N
31	F	35-45	5	3	25	26	N
44	F	35-45	58	43	0	0	N
47	F	65-75	21	4	9	6	N
61	F	65-75	40	95	8	6	N
71	F	15-25	24	21	0	6	N
74	F	65-75	31	6	10	6	N
77	F	75-85	68	25	11	6	N
79	F	55-65	32	2	15	0	N
1	M	75-85	33	18	189	160	Y
2	F	65-75	47	2	19	300	Y
3	M	75-85	35	15	1098	300	Y
4	M	65-75	17	7	128	300	Y
5	M	35-45	12	3	5	200	Y
6	F	55-65	26	12	68	60	Y
7	M	45-55	8	47	6	130	Y
8	M	65-75	15	3	45	114	Y
9	F	45-55	2	3	33	50	Y
10	M	45-55	8	3	100	340	Y
11	F	65-75	2	3	66	301	Y
16	F	45-55	17	8	18	340	Y
18	F	25-35	35	15	25	300	Y
20	F	65-75	15	3	172	38	Y
21	F	35-45	52	30	16	300	Y
22	F	35-45	24	6	29	300	Y
23	F	45-55	63	3	65	7	Y
27	F	35-45	86	9	53	150	Y
30	F	55-65	5	3	188	86	Y
36	F	45-55	21	12	36	340	Y
37	F	65-75	2	3	17	180	Y
38	F	35-45	29	11	45	300	Y
39	M	25-35	5	3	354	300	Y
40	F	25-35	8	3	64	180	Y
41	F	45-55	5	4	18	301	Y
42	F	25-35	8	6	159	340	Y
43	F	25-35	12	3	11	300	Y
45	F	25-35	5	3	35	341	Y
46	F	65-75	32	8	32	6	Y
48	F	45-55	5	3	121	340	Y
49	F	55-65	27	10	199	340	Y
50	F	55-65	48	45	251	301	Y
52	F	35-45	12	2	26	150	Y
53	F	55-65	38	25	73	301	Y
55	F	65-75	30	7	122	301	Y
56	F	35-45	9	2	23	49	Y
57	F	55-65	47	36	284	255	Y
58	F	45-55	2	3	73	301	Y
59	F	65-75	13	2	41	44	Y
62	F	75-85	16	2	79	301	Y
63	M	45-55	21	13	99	301	Y
66	F	65-75	8	2	263	301	Y
67	F	55-65	76	27	289	26	Y
68	F	55-65	5	11	5	204	Y
70	F	35-45	5	2	256	301	Y
72	M	55-65	20	2	489	301	Y
73	F	25-35	13	2	79	301	Y
78	F	35-45	27	11	18	301	Y
80	F	55-65	24	5	216	120	Y
81	F	45-55	19	2	79	180	Y
82	M	15-25	12	2	52	340	Y

Supplementary Table 3. Complete list of variants identified in the sorted Vb22 enriched CD8+ lymphocytes in the index patient. Variants were visually confirmed with IGV inspection and the variants with additional sanger sequencing confirmation are marked with **. Allelic frequencies (AF) at three time points (1, 2 and 3) are presented or the visually confirmed variants that were identified from the clonally expanded, v-beta restricted cell fraction.

Abbreviations:

3'UTR	3_prime_UTR_variant,NMD_transcript_variant
5'UTR	5_prime_UTR_variant
DS	downstream_gene_variant
IN	intron_variant
MS	missense_variant
SPL	missense_variant,splice_region_variant
SPL	splice_acceptor_variant
SPL	splice_donor_variant
STOP	stop_gained
SYN	synonymous_variant
NCE	non_coding_transcript_exon_variant
US	upstream_gene_variant

Gene	Chr	Pos	Ref	Alt	AF-1	AF-2	AF-3	aa-cha	Type	Exon
ZSWIM8	10	73796850	G	C		0,06	0,05	-	3'UTR	-
ZBED6	1	203799286	A	C	0,39	0,32	0,41	-	5'UTR	-
HIVEP3	1	41580664	G	T	0,37	0,39	0,43	-	DS	-
CYTH3	7	6165764	A	T		0,06	0,08	-	DS	-
XRRA1	11	74843418	C	A	0,08	0,02		-	DS	-
GPATCH4	1	156598282	C	A		0,05	0,12	-	DS	
OR51F1	11	4769849	C	A	0,39	0,37	0,44	-	IN	-
MARK4	19	45298162	A	C	0,36	0,27	0,39	-	IN	-
TRIM6	11	5611082	G	T	0,08	0,02		-	IN	-
CSMD1	8	2955672	G	C	0,08	0,03	0,03	P3305R	MS	64/70
UMAD1	7	7877353	G	C	0,38	0,38	0,41	-	MS	4/4
PLCB1	20	8371425	G	A	0,04	0,03	0,03	C74Y	MS	3/32
ZNF136	19	12187563	G	T	0,33	0,39	0,49	K395N	MS	4/4
MRC1	10	17827656	G	C	0,33	0,31	0,39	-	MS	3/30
PDHA1**	X	19349348	A	T	0,38	0,37	0,43	N32Y	MS	3/12
RAP1GAP	1	21603777	G	A	0,41	0,36	0,35	-	MS	16/23

ZNF100	19	21726719	T	G	0,34	0,36	0,47	-	MS	5/5
SEZ6L	22	26299011	G	A	0,36	0,30	0,47	R397H	MS	5/17
GABRA5	15	26943284	A	T	0,10	0,10	0,12	Y316F	MS	10/11
TAF12	1	28621951	G	C	0,08	0,02	0,02	P44R	MS	2/6
TEX15	8	30848407	G	C	0,05	0,03	0,03	S204C	MS	8/11
ITGAM	16	31275594	A	G	0,07	0,07	0,05	T302A	MS	9/30
AATF	17	36953084	C	G	0,04	0,11	0,08	T161S	MS	3/12
WDR87	19	37885428	A	G	0,39	0,37	0,45	L2748S	MS	6/6
SCN10A	3	38752256	G	C	0,39	0,40	0,44	P573R	MS	12/28
DNAH8	6	39008864	A	C	0,06	0,06	0,07	Q4205P	MS	89/93
GRAMD4	22	46661412	A	G	0,29	0,33	0,42	K146E	MS	5/19
SLC2A10	20	46726097	C	T	0,03	0,09	0,07	P354L	MS	2/5
CAB39L	13	49350794	C	T	0,30	0,36	0,37	V172M	MS	8/12
SIGLEC14	19	51646301	A	C	0,37	0,42	0,42	V126G	MS	2/7
TRAM2	6	52509576	A	G	0,06	0,06	0,07	L141S	MS	5/11
ZXDA	X	57908893	A	G	0,33	0,37	0,40	F510L	MS	1/1
LRIG3	12	58887870	A	G	0,02	0,03	0,12	L337P	MS	8/19
LRIG3	12	58887871	G	T	0,02	0,03	0,12	L337I	MS	8/19
TMEM30B	14	61280469	G	A	0,34	0,32	0,40	P227S	MS	1/1
KIAA1468	18	62258055	G	T	0,35	0,35	0,43	M668I	MS	14/30
AFTPH	2	64553013	G	C	0,31	0,38	0,44	L513F	MS	2/10
APOH	17	66214641	T	A	0,38	0,32	0,40	K265I	MS	7/8
UGT2B17	4	68537747	A	T	0,08	0,03	0,06	S491T	MS	6/6
EXD2	14	69228930	A	G	0,39	0,33	0,37	R25G	MS	3/9
ADAM21	14	70458243	G	C	0,05	0,04	0,06	Q248H	MS	2/2
SMN1,SMN2	5	70951986	A	G	0,42	0,40	0,41	N294D	MS	8/9
THSD4	15	71758004	G	C	0,33	0,37	0,44	A840P	MS	15/18
COG1	17	73201674	G	C	0,26	0,34	0,40	R616T	MS	7/14
SEC24C	10	73751152	A	G	0,34	0,32	0,42	T73A	MS	4/24
SCAPER	15	76800270	C	T	0,32	0,34	0,40	-	MS	7/32
MYCBP2	13	77098155	T	A	0,02	0,04	0,10	K2962I	MS	56/83
PLCG2**	16	81936318	G	A	0,38	0,38	0,40	-	MS	27/33
ZNF469	16	88433903	G	C	0,37	0,30	0,34	G2117R	MS	2/2
PLCE1	10	94324906	T	A	0,39	0,34	0,38	D2245E	MS	31/32
TRIM56	7	101087892	C	G	0,39	0,36	0,46	L194V	MS	3/3

DYNC1H1	14	102008329	A	G	0,41	0,34	0,42	Y1990C	MS	29/78
GSTO2	10	104278036	G	A	0,29	0,23	0,48	A96T	MS	4/7
C14orf79	14	104991604	T	C	0,36	0,41	0,41	I228T	MS	3/5
PIK3CG**	7	106905254	A	G	0,32	0,30	0,41	K1059R	MS	11/11
HECTD4	12	112248409	G	C	0,04	0,12	0,33	-	MS	26/76
TLR4	9	117713098	A	G	0,05	0,05	0,06	K324E	MS	3/3
NKAP**	X	119943370	C	A	0,36	0,35	0,45	R79L	MS	1/9
KPNA1**	3	122433765	G	C	0,29	0,36	0,45	F382L	MS	12/14
FAM102A	9	127945675	T	C	0,05	0,04	0,05	Y232C	MS	7/11
PCDHA1	5	140786786	G	T	0,36	0,40	0,39	A166S	MS	1/4
CPA3	3	148869036	A	C	0,36	0,44	0,40	Y89S	MS	3/11
ZNF775	7	150388480	G	C	0,32	0,33	0,44	G4R	MS	2/3
GRIA1	5	153647083	C	T	0,04	0,09	0,07	R126C	MS	3/16
GRIA1	5	153674602	C	A	0,40	0,33	0,42	R126C	MS	3/16
PMVK	1	154929088	T	A	0,32	0,29	0,41	K83I	MS	3/5
NOX3	6	155428993	T	G	0,45	0,44	0,36	K316Q	MS	9/14
KIRREL	1	158087761	C	G	0,41	0,35	0,37	P223R	MS	6/15
TLL1	4	165994510	A	G	0,04	0,02	0,03	Y164C	MS	4/21
SCN7A	2	166432676	G	T	0,02	0,03	0,03	A745E	MS	16/26
HNRNPH1**	5	179616162	C	G	0,33	0,42	0,51	G422R	MS	11/13
C4orf47	4	185445059	T	A	0,05	0,05	0,04	D270E	MS	6/7
CACNA1S	1	201049068	G	C	0,01	0,02	0,02	L1425V	MS	35/44
USH2A	1	216196664	G	C	0,08	0,14	0,27	I1380M	MS	19/72
HTR2B	2	231109322	A	G	0,03	0,03	0,08	F214S	MS	4/4
ITGAE**	17	3734894	T	C		0,36	0,47	N860D	MS	21/31
CAPZB	1	19356723	T	G	0,05		0,08	Y196S	MS	6/9
OSBPL1A	18	24178037	C	A		0,03	0,10	G690V	MS	21/28
NWD2	4	37444104	T	G	0,03		0,01	L706V	MS	7/7
WDR70	5	37702958	C	G	0,06	0,02		C429W	MS	13/18
ZNF621	3	40532426	A	C	0,06		0,01	N219T	MS	5/5
EP300	22	41129936	T	G	0,06	0,03		N405K	MS	5/31
TMEM72	10	44933677	G	A		0,05	0,11	A84T	MS	4/5
ZNF611	19	52705889	T	C	0,07	0,03		K389R	MS	6/6
SOCS4	14	55043960	A	G	0,06		0,01	T307A	MS	3/3
ADGRL3	4	62070184	A	C	0,04		0,02	Y1229S	MS	25/25

OPHN1	X	68274783	A	C	0,02	0,04		I113M	MS	5/25
BEX4	X	103216202	G	C	0,01	0,03		A17P	MS	3/3
GPR85	7	113083805	T	C		0,34	0,40	Y306C	MS	3/3
PROZ	13	113163083	A	C		0,04	0,08	T112P	MS	5/9
ALAD	9	113390854	T	A	0,03	0,02		N114I	MS	5/12
ZHX2	8	122952313	A	T	0,06	0,03		N268I	MS	3/4
ATP6V0A2	12	123727851	A	T		0,04	0,02	K197I	MS	6/20
GOLGA3	12	132804851	G	C	0,04		0,15	Q488E	MS	7/24
TRPV5	7	142912553	A	C	0,08		0,03	L573V	MS	13/15
TTN	2	178566426	A	G	0,05	0,02		V24928A	MS	326/363
PTPRC	1	198756083	A	G	0,05	0,03		-	MS	33/33
C1orf116	1	207023069	G	C		0,04	0,09	T232R	MS	4/4
EIF3B	7	2360747	A	C	0,04			E179D	MS	2/19
PIK3CD	1	9716498	C	T			0,02	A220V	MS	6/24
MKRN3	15	23566815	C	T			0,03	-	MS	1/1
KIAA1217	10	24545884	C	G	0,08			P1798A	MS	21/21
PLAUR	19	43649088	T	A	0,05			Q270H	MS	7/7
CYP4X1	1	47048573	G	A			0,11	-	MS	10/12
PKD1L1	7	47866601	G	C	0,39			L1304V	MS	25/57
MFSD5	12	53253247	G	A	0,06			G138R	MS	2/2
WNK3	X	54293322	T	C	0,05			N567D	MS	9/24
TENM4	11	78701568	A	T			0,03	L1682Q	MS	28/34
AFF3	2	99551497	G	A			0,02	R1220W	MS	25/25
SELPLG	12	108623632	T	G	0,09			-	MS	2/2
SLC2A12	6	134029685	G	A	0,03			A47V	MS	2/5
ERICH6	3	150666978	G	C	0,04			Q513E	MS	13/14
ADAR	1	154601651	T	G	0,08			N331H	MS	2/15
SMC4	3	160402775	A	G	0,06			K140E	MS	3/23
FASTKD2	2	206766703	A	T	0,07			T4S	MS	2/12
SLC12A3	16	56869729	T	A	0,01	0,01	0,02	V169D	SPL	4/26
MMS22L**	6	97267873	T	C	0,37	0,37	0,46	K276R	SPL	8/25
DCDC1	11	31305721	G	A	0,03	0,06	0,09	-	NCE	
MUC19	12	40450266	A	C	0,19	0,33	0,27	-	NCE	42/173
MAP4	3	47928352	T	G	0,07	0,09	0,10	-	SPL	-
LAMA4	6	112178233	C	G	0,36	0,38	0,50	-	SPL	-

MED12L	3	151416311	G	C	0,50	0,28	0,36	-	SPL	-
C5orf42	5	37120214	A	G	0,03	0,05	0,10	-	SPL	-
COL3A1	2	188994596	T	G	0,36	0,34	0,46	-	SPL	-
NKAPL	6	28259429	C	T	0,44	0,42	0,46	-	STOP	1/1
CCND1	11	69641416	G	T	0,47	0,34	0,31	-	STOP	1/5
PPM1D	17	60663448	C	T	0,05	0,03		-	STOP	6/6
AGA	4	177437464	A	C	0,04		0,03	-	STOP	5/9
OR1E1	17	3398303	A	G	0,04	0,02	0,40	-	SYN	1/1
NAGPA	16	5025604	G	A	0,33	0,40	0,42	-	SYN	10/10
ACTB	7	5528378	G	C	0,48	0,37	0,33	-	SYN	4/6
GRPEL1	4	7064136	C	T	0,30	0,35	0,39	-	SYN	2/4
TMPRSS15	21	18343632	G	C	0,35	0,40	0,45	-	SYN	12/25
MPP7	10	28054112	A	G	0,49	0,32	0,41	-	SYN	18/18
PSMD11	17	32469066	A	G	0,08	0,12	0,15	-	SYN	6/14
NFX1	9	33294526	T	C	0,34	0,39	0,42	-	SYN	2/24
NOL6	9	33467436	C	A	0,36	0,37	0,39	-	SYN	13/26
SPTBN1	2	54629705	G	A	0,36	0,41	0,40	-	SYN	14/36
LRIG3	12	58880759	C	A	0,02	0,03	0,02	-	SYN	13/19
GATA5	20	62466507	C	A	0,36	0,35	0,40	-	SYN	4/7
DNA2	10	68422378	A	G	0,06	0,06	0,05	-	SYN	17/21
NEGR1	1	71611064	C	T	0,05	0,08	0,06	-	SYN	5/7
SHQ1	3	72750353	G	C	0,36	0,50	0,39	-	SYN	11/11
GNAT3	7	80458794	T	C	0,07	0,05	0,11	-	SYN	8/8
GPAT3	4	83596861	A	G	0,30	0,35	0,32	-	SYN	8/12
CYP3A7-CYP3A51P	7	99695804	A	G	0,24	0,39	0,48	-	SYN	14/15
CCDC168	13	102731626	A	G	0,01	0,01	0,03	-	SYN	4/4
ASTN2	9	116725853	G	C	0,04	0,02	0,04	-	SYN	15/22
BBS7	4	121853034	T	C	0,41	0,35	0,42	-	SYN	8/19
LAMC3	9	131049090	C	G	0,33	0,42	0,39	-	SYN	9/28
RSPH3	6	158999659	C	T	0,37	0,40	0,38	-	SYN	1/8
SLC22A2	6	160245465	T	C	0,37	0,40	0,44	-	SYN	6/11
COBLL1	2	164722213	C	T	0,34	0,37	0,38	-	SYN	9/17
FZD5	2	207768662	G	C	0,32	0,42	0,47	-	SYN	2/2
RBM44	2	237818083	G	T	0,31	0,36	0,40	-	SYN	3/16
MUC6	11	1026075	G	A	0,05		0,04	-	SYN	21/33

HSPH1	13	31151702	G	A	0,07		0,03	-	SYN	6/18
SLC28A2	15	45272689	C	T		0,33	0,46	-	SYN	17/18
NRXN1	2	51028061	G	A		0,02	0,03	-	SYN	2/24
DSCAML1	11	117525016	G	C	0,04	0,03		-	SYN	5/33
HDDC2	6	125298798	C	T	0,09		0,02	-	SYN	3/6
MEIKIN	5	131818795	A	G	0,09	0,03		-	SYN	12/13
KIF2A	5	62363243	A	G	0,07			-	SYN	13/21
HEXA	15	72345472	C	T	0,30	0,41	0,53	-	SYN	
RXRB	6	33200365	G	C	0,37	0,46	0,48	-	US	-
CDC45	22	19483870	A	T	0,04	0,11		-	US	-
OTOP2	17	74927250	G	C		0,03	0,06	-	US	-
ANAPC15	11	72107368	T	A	0,04			-	US	-
